# Design, Synthesis and Bioactivities of Novel 1,4-Pentadien-3-one Derivatives Containing a Substituted Pyrazolyl Moiety

**DOI:** 10.3390/molecules22071126

**Published:** 2017-07-06

**Authors:** Cuili Chen, Jia Chen, Haiying Gu, Ning Bao, Hong Dai

**Affiliations:** 1 College of Chemistry, Chemical Engineering and Materials Science, Soochow University, Suzhou 215123, China; cleancucumber@163.com; 2 School of Public Health, Nantong University, Nantong 226019, China; ningb2000@yahoo.com; 3 College of Chemistry and Chemical Engineering, Nantong University, Nantong 226019, China; 15642891665@163.com

**Keywords:** penta-1,4-dien-3-one, pyrazole, synthesis, biological activity

## Abstract

In this study, in order to find novel biologically active penta-1,4-dien-3-one derivatives, a series of penta-1,4-dien-3-one compounds containing a substituted pyrazole subunit were designed and synthesized. Their structures were characterized by ^1^H-NMR, ^13^C-NMR and elemental analysis. The preliminary bioassays displayed that most of the title compounds showed significant antiproliferative activity against HepG2 cell lines. Especially, compounds **7a**–**m**, **o**, **r**, **s**, **u**, **w**, **y** and **z** were active against HepG2 cells with IC_50_ values of 0.10–5.05 μM, which were superior to that of the contrast sorafenib (IC_50_ = 16.20 μM).

## 1. Introduction

**Figure 1:**
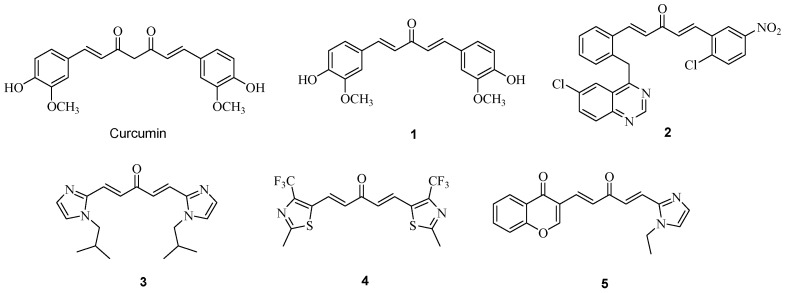
Structures of curcumin and curcumin analogues/mimics.

In the past few decades, curcumin, the principal curcuminoid of turmeric and its derivatives have attracted considerable attention due to their remarkable spectrum of biological activity, such as antimicrobial [1], anticancer [2], antioxidant [3], anti-inflammatory [4] and anti-HIV [5] activities. Moreover, curcumin exhibits therapeutic promise for prostate cancer by interfering with cell proliferation through the induction of cell cycle arrest [6]. Curcumin is a potent anticancer agent with remarkable safety profile [7]. Despite that, accumulating evidence suggests that curcumin is highly unstable and has a poor bioavailability when tested in vitro and in vivo [8]. This has triggered extensive research in search for curcumin analogues and mimics with improved potency and pharmacokinetic profiles for the potential clinical treatment of cancers. Recently, many curcumin analogues have been studied for their activities by chemically modifying curcumin [9,10,11,12,13,14]. Curcumin mimic **1** (Figure 1) showed 5–5.5-fold increase in the cytotoxic potency relative to curcumin towards PC-3 cell line by using a five-carbon dienone linear linker instead of a seven-carbon β-diketone linker in curcumin [15]. Further, curcumin analogues have been more well documented for their enhanced potency than curcumin, when imidazoles (Figure 1) [16], quinazoline (Figure 1) [17], thiazole (Figure 1) [18] or chromone (Figure 1) [19] moieties were introduced into curcumin mimic **1**. From these observations, it can be noticed that penta-1,4-dien-3-one is an optimal scaffold for developing curcumin mimics as potential antitumor agents. It is therefore very meaningful to profound investigation of curcumin mimics as potential drug candidates for the treatment of cancer.

**Figure 2:**
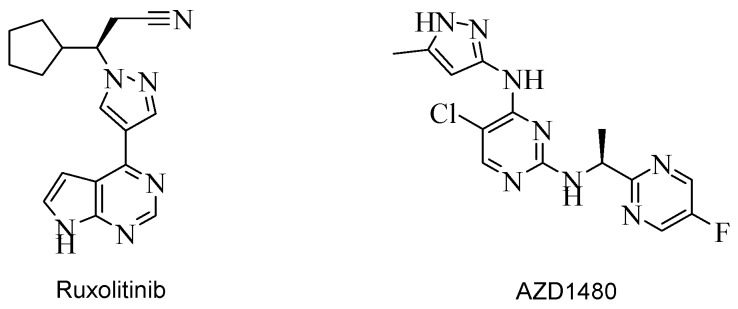
Structures of Ruxolitinib and AZD1480.

On the other hand, literature survey revealed that some pyrazole derivatives have received extensive attention owing to their synthetic and biological importance. Compounds attaching this structural unit exhibit diverse biological activities such as antileukemic [20,21], antitumor [22,23], and anti-proliferative [24,25] activities. Therapeutically interesting drug candidates: for example, Ruxolitinib and AZD1480 (Figure 2), are known to contain an important pyrazole nucleus [26,27].

Furthermore, literatures have previously reported that the pharmacological attributes of curcumin improved when pyrazole unit was attached to curcumin [28,29]. Taking into account the structural relevance of this class of curcumin and pyrazole analogues, we hypothesized whether the introduction of pyrazole moiety into curcumin has any effect against cancer cells.

**Figure 3:**
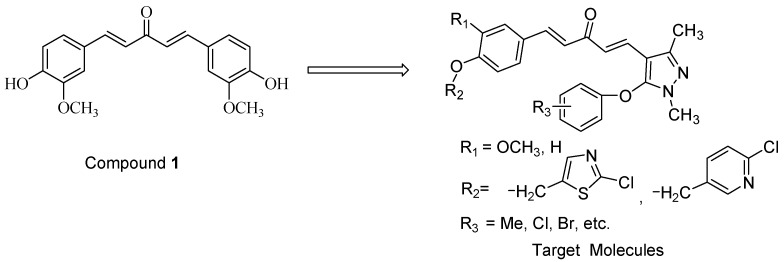
The design of the target molecules.

In this context, we desire to probe into the pharmacological potential of novel curcumin analogues containing substituted pyrazole moiety (Figure 3) based on pharmacophores hybridization. It was thought that these novel skeletons bearing two pharmacological groups such as penta-1,4-dien-3-one and pyrazole would be well synergistic antitumor effects. Herein, we report the synthesis of a series of novel penta-1,4-dien-3-one derivatives containing pyrazole ring and evaluated for their antitumor activity. It was expected that higher synergistic effect could be achieved by chemical modifications of R_1_, R_2_ and R_3_ on the benzene ring or pyrazole ring, respectively.

## 2. Results and Discussion

### 2.1. Chemistry

**Scheme 1:**
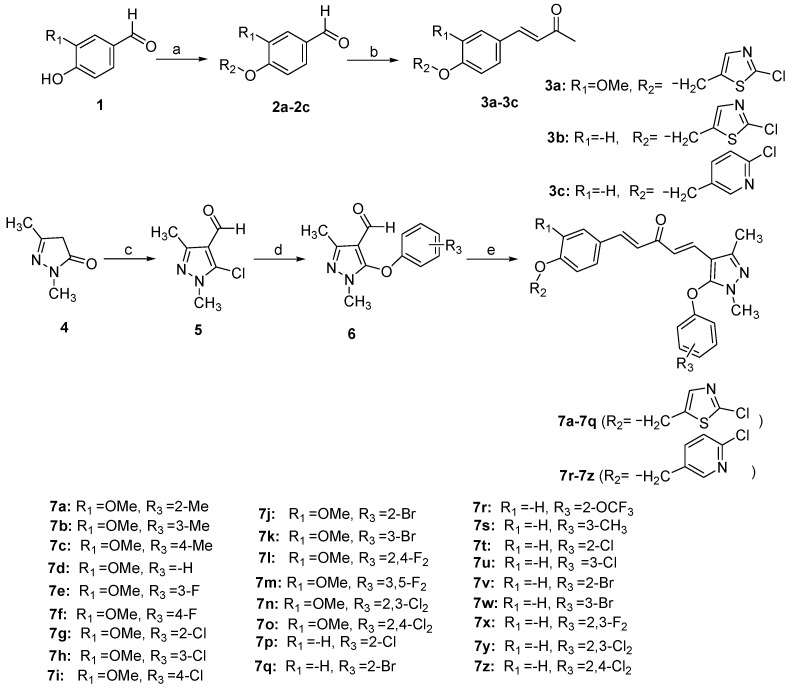
Synthesis of the target compounds **7a**–**7z**. *Reagents and conditions:* (a) 2-chloro-5-chloromethylthiazole or 2-chloro-5-chloromethylpyridine, potassium carbonate, ethanol, reflux for 8–10 h, 72% for **2a**, 68% for **2b**, 75% for **2c**; (b) acetone, 10% sodium hydroxide, r.t. for 12–15 h, 70% for **3a**, 67% for **3b**, 77% for **3c**; (c) *N*,*N*-dimethylformamide, POCl_3_, 0 °C for 30 min, then 110 °C for 10 h, 80% for **5**; (d) phenols, potassium hydroxide, DMF, 40 °C for 6 h, then 100 °C for 11–22 h, 55–82% for **6**; (e) compound **3**, 10% sodium hydroxide, ethanol, rt. for 10–15 h, 50–72% for **7**.

As shown in Scheme 1, 26 penta-1,4-dien-3-one compounds bearing a substituted pyrazole moiety were successfully synthesized. Using potassium carbonate as the base, compound **1** was easily reacted with 2-chloro-5-chloromethylthiazole or 2-chloro-5-chloromethylpyridine to provide compound **2**. Further reaction with acetone under basic condition gave compound **3** in good yields. Compound **4** was subjected to the Vilsmeier–Haack reaction using a mixture of *N*,*N*-dimethylformamide and POCl_3_ to produce 1,3-dimethyl-5-chloro-4-formylpyrazole (**5**) smoothly. The treatment of compound **5** with various substituted phenols under basic conditions afforded 1,3-dimethyl-5-aryloxy-4-formyl pyrazole (**6**). Finally, the reaction of the key intermediate **3** with compound **6** under sodium hydroxide-promoting conditions formed the target compounds **7a**–**7z** in good yields. The structures of the title compounds were characterized by ^1^H-NMR, ^13^C-NMR and elemental analysis. 

### 2.2. Biological Activities

The title compounds **7a**–**7z** were screened for their cancer cell growth inhibitory activity against human gastric cancer cells (SGC-7901), human hepatoma cells (HepG2) and human pancreatic carcinoma cells (Panc-1) in vitro by the 3-(4,5-dimethylthiazol-2-yl)-2,5-diphenyltetrazolium bromide (MTT) assay. Sorafenib was selected as the positive control. The IC_50_ values of compounds **7a**–**7z** against SGC-7901, HepG2 and Panc-1 cells were listed in Table 1. As can be seen, most of the target compounds displayed more potent antiproliferative activity on HepG2 cells than SGC-7901 and Panc-1 cells. The IC_50_ values of **7a**–**m**, **o**, **r**, **s**, **u**, **w**, **y** and **z** against HepG2 cell lines were 0.10–5.05 μM, respectively, which were much better than that of the control sorafenib (IC_50_ = 16.20 μM). Based on the structure–activity data, we can find that when R_2_ is 2-chlorothiazole-5-methylene and R_1_ is OMe, 3-methyl-substituted compound **7b** and 2-chloro-substituted compound **7g** showed more potent inhibitory activity against HepG2 cell than other analogues, and when R_2_ is 2-chlorothiazole-5-methylene and R_3_ is 2-chloro or 2-bromo, 3-methoxy-substituted compound **7g** or **7j** exhibited better antiproliferative activity than did the corresponding unsubstituted derivative **7p** or **7q**. Moreover, we can also see that when R_2_ is 2-chloropyridine-5-methylene (R_1_ = H), the substituent at 3-position of phenyl ring was halogen (**7u** and **7w**) or the substituents at 2,4-position of phenyl ring were chloro atoms (**7z**), it was advantageous to increase the antiproliferative property against HepG2 cell lines. 

All the above results provided important data for the design and development of penta-1,4-dien-3-one derivatives as novel antiproliferative agents in future. Additional structural optimization and biological evaluations are ongoing in our laboratory.

## 3. Experimental Section

### 3.1. Chemistry

#### 3.1.1. General Procedures

All reagents are commercially available and used without further purification except as indicated. The melting points were determined on an X-4 binocular microscope melting point apparatus (Beijing Tech Instrument Co., Beijing, China) and are uncorrected. ^1^H-NMR and ^13^C-NMR spectra were measured on a Bruker AV400 spectrometer (400 MHz, ^1^H; 100 MHz, ^13^C, Bruker, Billerica, MA, USA) in CDCl_3_ or DMSO-*d*_6_ with TMS as an internal standard. Elemental analyses were determined on a Yanaco CHN Corder MT-3 elemental analyzer (Yanaco, Kyoto, Japan). The intermediate 1-methyl-3-methyl-*1H*-pyrazol-5-one (**4**) was prepared according to reported procedures [30,31].

#### 3.1.2. Synthesis of Compound **2**

To a solution of 2-chloro-5-chloromethylthiazole (or 2-chloro-5-chloromethylpyridine) (20 mmol), compound **1** (22 mmol) and anhydrous ethanol (80 mL) was added potassium carbonate (24 mmol) at room temperature. The resulting mixture was heated to reflux for 8–10 h. Then the solvent was concentrated in rotatory evaporator to give brown solid, which was washed by 15% sodium hydrate solution, dried and recrystallized from ethanol to produce compound **2**. Compound **2a**: yellow solid, yield 72%, m.p. 118–120 °C. ^1^H-NMR (CDCl_3_): δ 9.88 (s, 1H, CHO), 7.58 (s, 1H, Thiazole-H), 7.43–7.46 (m, 2H, Ar-H), 7.05 (d, *J* = 8.8 Hz, 1H, Ar-H), 5.32 (s, 2H, CH_2_), 3.94 (s, 3H, OCH_3_); ^13^C-NMR (CDCl_3_): δ 190.8, 153.3, 152.3, 150.4, 140.7, 135.4, 131.4, 126.2, 113.4, 109.9, 63.6, 56.1. Anal. calcd. for C_12_H_10_ClNO_3_S: C 50.80; H 3.55; N 4.94. Found: C 50.71; H 3.67; N 4.85. Compound **2b**: yellow solid, yield 68%, m.p. 103–105 °C. ^1^H-NMR (CDCl_3_): δ 9.91 (s, 1H, CHO), 7.87 (d, *J* = 8.4 Hz, 2H, Ar-H), 7.59 (s, 1H, Thiazole-H), 7.06 (d, *J* = 8.8 Hz, 2H, Ar-H), 5.27 (s, 2H, CH_2_); ^13^C-NMR (CDCl_3_): δ 190.6, 162.4, 153.2, 140.5, 135.2, 132.1, 130.8, 115.1, 62.6. Anal. calcd. for C_11_H_8_ClNO_2_S: C 52.08; H 3.18; N 5.52. Found: C 51.96; H 3.29; N 5.60. Compound **2c**: yellow solid, yield 75%, m.p. 113–115 °C. ^1^H-NMR (CDCl_3_): δ 9.91 (s, 1H, CHO), 8.48 (d, *J* = 2.0 Hz, 1H, Py-H), 7.86 (d, *J* = 8.4 Hz, 2H, Ar-H), 7.77 (dd, *J*_1_ = 2.4 Hz, *J*_2_ = 8.0 Hz, 1H, Py-H), 7.39 (d, *J* = 8.4 Hz, 1H, Py-H), 7.08 (d, *J* = 8.4 Hz, 2H, Ar-H), 5.15 (s, 2H, CH_2_); ^13^C-NMR (CDCl_3_): δ 190.7, 163.0, 151.6, 148.8, 138.1, 132.1, 130.6, 130.5, 124.4, 115.0, 66.9. Anal. calcd. for C_13_H_10_ClNO_2_: C 63.04; H 4.07; N 5.66. Found: C 63.17; H 4.01; N 5.75.

#### 3.1.3. Synthesis of Compound **3**

To a mixture of compound **2** (20 mmol) and acetone (50 mL) was added dropwise a solution of 10% sodium hydrate (24 mmol) at room temperature. The reaction mixture was stirred for another 12–15 h at room temperature. The resulting precipitate was collected by filtration, and the crude product was recrystallized from anhydrous ethanol to give compound **3**. Compound **3a**: yellow solid, yield 70%, m.p. 127–129 °C. ^1^H-NMR (CDCl_3_): δ 7.54 (s, 1H, Thiazole-H), 7.45 (d, *J* = 16.0 Hz, 1H, C=C-H), 6.92–7.09 (m, 3H, Ar-H), 6.62 (d, *J* = 16.0 Hz, 1H, C=C-H), 5.27 (s, 2H, CH_2_), 3.91 (s, 3H, OCH_3_), 2.38 (s, 3H, CH_3_); ^13^C-NMR (CDCl_3_): δ 198.3, 153.1, 150.3, 149.1, 143.0, 140.5, 136.0, 129.2, 126.0, 122.4, 115.0, 110.7, 63.8, 56.0, 27.4. Anal. calcd. for C_15_H_14_ClNO_3_S: C 55.64; H 4.36; N 4.33. Found: C 55.53; H 4.47; N 4.25. Compound **3b**: yellow solid, yield 67%, m.p. 112–114 °C. ^1^H-NMR (CDCl_3_): δ 7.56 (s, 1H, Thiazole-H), 7.52 (d, *J* = 8.8 Hz, 2H, Ar-H), 7.47 (d, *J* = 16.4 Hz, 1H, C=C-H), 6.97 (d, *J* = 8.8 Hz, 2H, Ar-H), 6.62 (d, *J* = 16.4 Hz, 1H, C=C-H), 5.21 (s, 2H, CH_2_), 2.37 (s, 3H, CH_3_); ^13^C-NMR (CDCl_3_): δ 198.3, 159.5, 153.0, 142.7, 140.3, 135.7, 130.0, 128.2, 125.7, 115.3, 62.6, 27.5. Anal. calcd. for C_14_H_12_ClNO_2_S: C 57.24; H 4.12; N 4.77. Found: C 57.11; H 4.24; N 4.86. Compound **3c**: yellow solid, yield 77%, m.p. 128–130 °C. ^1^H-NMR (CDCl_3_): δ 8.46 (d, *J* = 2.4 Hz, 1H, Py-H), 7.76 (dd, *J*_1_ = 2.4 Hz, *J*_2_ = 8.0 Hz, 1H, Py-H), 7.56 (d, *J* = 8.8 Hz, 2H, Ar-H), 7.47 (d, *J* = 16.4 Hz, 1H, C=C-H), 7.38 (d, *J* = 8.4 Hz, 1H, Py-H), 6.98 (d, *J* = 8.8 Hz, 2H, Ar-H), 6.62 (d, *J* = 16.4 Hz, 1H, C=C-H), 5.10 (s, 2H, CH_2_), 2.37 (s, 3H, CH_3_); ^13^C-NMR (CDCl_3_): δ 198.3, 160.0, 151.4, 148.8, 142.8, 138.1, 131.0, 130.1, 128.0, 125.5, 124.4, 115.2, 66.8, 27.5. Anal. calcd. for C_16_H_14_ClNO_2_: C 66.79; H 4.90; N 4.87. Found: C 66.90; H 4.82; N 4.96.

#### 3.1.4. Synthesis of Compound **5**

To a well-stirred cold (0 °C) solution of *N*, *N*-dimethylformamide (20 mmol) was added dropwise POCl_3_ (30 mmol). Compound **4** (20 mmol) was added to the above reaction mixture in portions. The resulting mixture was stirred at 0 °C for 30 min and then heated to 110 °C for 10 h. The reaction solution was cooled to room temperature and poured into ice-water. The pH value was adjusted to 7–8 by sodium hydroxide solution, and the yellow solid was combined by filtration. The filtrate was continuously extracted with ethyl acetate (3 × 80 mL). The combined organic layer was dried over anhydrous Na_2_SO_4_, and concentrated in rotatory evaporator to afford yellow solid. The solid obtained in two portions was gathered together and then was recrystallized from petroleum ether/ethyl acetate to produce compound **5**, yield 80%, m.p. 76–78 °C. ^1^H-NMR (CDCl_3_): δ 9.86 (s, 1H, CHO), 3.82 (s, 3H, N-CH_3_), 2.45 (s, 3H, CH_3_); ^13^C-NMR (CDCl_3_): δ 183.5, 150.9, 133.7, 116.3, 35.9, 13.6.

#### 3.1.5. General Procedure for the Preparation of Compound **6**

To a well-stirred solution of substituted phenol (30 mmol) in *N*,*N*-dimethylformamide (50 mL) was added potassium hydroxide (40 mmol) at room temperature. The resulting mixture was warmed to 40 °C for 6 h, and then intermediate **5** (20 mmol) was added thereto. The resulting mixture was heated to 100 °C for 11–22 h. The reaction solution was cooled to room temperature, poured into ice-water (80 mL). The reaction mixture was extracted with ethyl acetate (3 × 30 mL) and the organic layer was dried over anhydrous sodium sulfate. The solvent was removed under reduced pressure to provide intermediates **6** in yields ranging from 55% to 82% [32].

#### 3.1.6. General Procedure for the Preparation of Compounds **7a**–**7z**

To a well-stirred solution of intermediate **3** (5 mmol) and compound **6** (6 mmol) in ethanol (25 mL) was added dropwise a solution of 10% sodium hydroxide (8 mmol) at room temperature. The resulting mixture was stirred at room temperature for 10–15 h. The reaction mixture was filtered to afford the crude product, which was then subjected to column chromatography using a mixture of petroleum ether and ethyl acetate as an eluent to produce the target compounds **7a**–**7z**, with yields ranging from 50% to 72%. All 26 penta-1,4-dien-3-one derivatives **7a**–**7z** were novel and the physical and spectral data for these compounds are listed below. ^1^H-NMR and ^13^C-NMR spectra are provided in the Supplementary Materials.

*1-{4-[(2-Chlorothiazol-5-yl)methoxy]-3-methoxyphenyl}-5-[5-(2-methylphenoxy)-1,3-dimethyl-1H-pyrazol-4-yl]penta-1,4-dien-3-one* (**7a**). Yellow oil, yield 58%. ^1^H-NMR (CDCl_3_): δ 7.53 (s, 1H, Thiazole-H), 6.52–7.47 (m, 11H, 2 × CH=CH and Ar-H), 5.25 (s, 2H, CH_2_), 3.91 (s, 3H, OCH_3_), 3.60 (s, 3H, N-CH_3_), 2.49 (s, 3H, CH_3_), 2.42 (s, 3H, CH_3_); ^13^C-NMR (CDCl_3_): δ 188.7, 154.1, 153.0, 150.3, 148.9, 148.5, 141.8, 140.4, 136.1, 132.0, 131.8, 129.9, 127.6, 126.4, 125.5, 123.8, 122.1, 121.9, 115.3, 113.3, 111.1, 104.2, 64.0, 56.0, 34.3, 16.1, 14.0. Anal. calcd. for C_28_H_26_ClN_3_O_4_S: C 62.74; H 4.89; N 7.84. Found: C 62.63; H 4.98; N 7.96.

*1-{4-[(2-Chlorothiazol-5-yl)methoxy]-3-methoxyphenyl}-5-[5-(3-methylphenoxy)-1,3-dimethyl-1H-pyrazol-4-yl]penta-1,4-dien-3-one* (**7b**). Yellow solid, yield 64%, m.p. 158–160 °C. ^1^H-NMR (DMSO-*d*_6_): δ 7.80 (s, 1H, Thiazole-H), 6.64–7.42 (m, 11H, 2 × CH=CH and Ar-H), 5.37 (s, 2H, CH_2_), 3.83 (s, 3H, OCH_3_), 3.57 (s, 3H, N-CH_3_), 2.37 (s, 3H, CH_3_), 2.30 (s,3H, Ar-CH_3_); ^13^C-NMR (DMSO-*d*_6_): δ 188.1, 156.2, 151.7, 149.9, 149.4, 148.2, 147.7, 142.4, 141.8, 140.8, 137.3, 131.7, 130.6, 129.1, 125.2, 123.2, 122.8, 116.1, 114.6, 112.6, 111.8, 103.7, 99.3, 63.0, 56.3, 34.6, 21.4, 14.2. Anal. calcd. for C_28_H_26_ClN_3_O_4_S: C 62.74; H 4.89; N 7.84. Found: C 62.87; H 4.77; N 7.73.

*1-{4-[(2-Chlorothiazol-5-yl)methoxy]-3-methoxyphenyl}-5-[5-(4-methylphenoxy)-1,3-dimethyl-1H-pyrazol-4-yl]penta-1,4-dien-3-one* (**7c**). Yellow solid, yield 72%, m.p. 148–150 °C. ^1^H-NMR (DMSO-*d*_6_): δ 7.80 (s, 1H, Thiazole-H), 6.63–7.42 (m, 11H, 2 × CH=CH and Ar-H), 5.37 (s, 2H, CH_2_), 3.83 (s, 3H, OCH_3_), 3.56 (s, 3H, N-CH_3_), 2.37 (s, 3H, CH_3_), 2.26 (s, 3H, Ar-CH_3_); ^13^C-NMR (DMSO-*d*_6_): δ 188.1, 154.2, 151.7, 149.9, 149.3, 148.4, 147.7, 142.4, 141.8, 137.3, 133.5, 131.7, 131.2, 129.1, 125.0, 123.2, 122.9, 115.5, 114.5, 111.7, 103.7, 62.9, 56.2, 34.6, 20.6, 14.2. Anal. calcd. for C_28_H_26_ClN_3_O_4_S: C 62.74; H 4.89; N 7.84. Found: C 62.81; H 5.01; N 7.77.

*1-{4-[(2-Chlorothiazol-5-yl)methoxy]-3-methoxyphenyl}-5-(5-phenoxy-1,3-dimethyl-1H-pyrazol-4-yl)penta-1,4-dien-3-one* (**7d**). Yellow solid, yield 66%, m.p. 138–140 °C. ^1^H-NMR (DMSO-*d*_6_): δ 7.80 (s, 1H, Thiazole-H), 6.63–7.46 (m, 12H, 2 × CH=CH and Ar-H), 5.37 (s, 2H, CH_2_), 3.83 (s, 3H, OCH_3_), 3.58 (s, 3H, N-CH_3_), 2.37 (s, 3H, CH_3_); ^13^C-NMR (DMSO-*d*_6_): δ 188.1, 156.2, 151.7, 149.9, 149.3, 148.1, 147.7, 142.3, 141.8, 137.3, 131.6, 130.9, 129.1, 125.0, 124.5, 123.1, 122.9, 115.7, 114.6, 111.8, 103.7, 63.0, 56.3, 34.6, 14.2. Anal. calcd. for C_27_H_24_ClN_3_O_4_S: C 62.12; H 4.63; N 8.05. Found: C 62.01; H 4.75; N 8.15.

*1-{4-[(2-Chlorothiazol-5-yl)methoxy]-3-methoxyphenyl}-5-[5-(3-fluorophenoxy)-1,3-dimethyl-1H-pyrazol-4-yl]penta-1,4-dien-3-one* (**7e**). Yellow oil, yield 55%. ^1^H-NMR (DMSO-*d*_6_): δ 7.80 (s, 1H, Thiazole-H), 6.64–7.44 (m, 11H, 2 × CH=CH and Ar-H), 5.37 (s, 2H, CH_2_), 3.83 (s, 3H, OCH_3_), 3.60 (s, 3H, N-CH_3_), 2.38 (s, 3H, CH_3_); ^13^C-NMR (DMSO-*d*_6_): δ 188.1, 163.4 (d, *J* = 244 Hz), 157.2, 157.1, 151.7, 149.9, 147.9, 147.6, 142.5, 141.8, 137.3, 132.4, 132.3, 131.4, 129.0, 125.0, 123.1 (d, *J* = 14 Hz), 114.5, 111.8, 111.6, 111.4, 104.2 (d, *J* = 26 Hz), 103.7, 62.9, 56.2, 34.7, 14.2. Anal. calcd. for C_27_H_23_ClFN_3_O_4_S: C 60.05; H 4.29; N 7.78. Found: C 60.18; H 4.21; N 7.66.

*1-{4-[(2-Chlorothiazol-5-yl)methoxy]-3-methoxyphenyl}-5-[5-(4-fluorophenoxy)-1,3-dimethyl-1H-pyrazol-4-yl]penta-1,4-dien-3-one* (**7f**). Yellow solid, yield 58%, m.p. 138–139 °C. ^1^H-NMR (DMSO-*d*_6_): δ 7.81 (s, 1H, Thiazole-H), 6.63–7.44 (m, 11H, 2 × CH=CH and Ar-H), 5.37 (s, 2H, CH_2_), 3.83 (s, 3H, OCH_3_), 3.59 (s, 3H, N-CH_3_), 2.37 (s, 3H, CH_3_); ^13^C-NMR (DMSO-*d*_6_): δ 188.1, 158.2 (d, *J* = 241 Hz), 152.3, 151.6, 149.9, 149.4, 148.3, 147.7, 142.4, 141.8, 137.3, 131.5, 129.1, 125.0, 117.5, 117.3, 114.6, 111.8, 63.0, 56.2, 34.7, 14.2. Anal. calcd. for C_27_H_23_ClFN_3_O_4_S: C 60.05; H 4.29; N 7.78. Found: C 59.92; H 4.41; N 7.91.

*1-{4-[(2-Chlorothiazol-5-yl)methoxy]-3-methoxyphenyl}-5-[5-(2-chlorophenoxy)-1,3-dimethyl-1H-pyrazol-4-yl]penta-1,4-dien-3-one* (**7g**). Yellow solid, yield 50%, m.p. 176–178 °C. ^1^H-NMR (DMSO-*d*_6_): δ 7.80 (s, 1H, Thiazole-H), 6.62–7.50 (m, 11H, 2 × CH=CH and Ar-H), 5.37 (s, 2H, CH_2_), 3.83 (s, 3H, OCH_3_), 3.58 (s, 3H, N-CH_3_), 2.37 (s, 3H, CH_3_); ^13^C-NMR (DMSO-*d*_6_): δ 188.1, 155.1, 151.8, 150.0, 149.5, 147.9, 147.8, 142.6, 142.0, 137.4, 131.5, 130.9, 129.2, 128.4, 125.0, 123.3, 123.2, 117.7, 114.6, 111.9, 103.8, 63.0, 56.3, 34.8, 14.3. Anal. calcd for C_27_H_23_Cl_2_N_3_O_4_S: C 58.28; H 4.17; N 7.55. Found: C 58.40; H 4.06; N 7.45.

*1-{4-[(2-Chlorothiazol-5-yl)methoxy]-3-methoxyphenyl}-5-[5-(3-chlorophenoxy)-1,3-dimethyl-1H-pyrazol-4-yl]penta-1,4-dien-3-one* (**7h**). Yellow oil, yield 54%. ^1^H-NMR (DMSO-*d*_6_): δ 7.80 (s, 1H, Thiazole-H), 6.63–7.47 (m, 11H, 2 × CH=CH and Ar-H), 5.37 (s, 2H, CH_2_), 3.83 (s, 3H, OCH_3_), 3.60 (s, 3H, N-CH_3_), 2.38 (s, 3H, CH_3_); ^13^C-NMR (DMSO-*d*_6_): δ 188.1, 156.8, 151.7, 149.9, 149.4, 147.9, 147.5, 142.5, 141.8, 137.3, 134.9, 132.4, 131.3, 129.0, 125.0, 124.7, 123.2, 123.1, 116.3, 114.5, 114.4, 111.8, 103.7, 62.9, 56.2, 34.7, 14.2. Anal. calcd. for C_27_H_23_Cl_2_N_3_O_4_S: C 58.28; H 4.17; N 7.55. Found: C 58.15; H 4.27; N 7.67.

*1-{4-[(2-Chlorothiazol-5-yl)methoxy]-3-methoxyphenyl}-5-[5-(4-chlorophenoxy)-1,3-dimethyl-1H-pyrazol-4-yl]penta-1,4-dien-3-one* (**7i**). Yellow oil, yield 62%. ^1^H-NMR (DMSO-*d*_6_): δ 7.80 (s, 1H, Thiazole-H), 6.61–7.70 (m, 11H, 2 × CH=CH and Ar-H), 5.37 (s, 2H, CH_2_), 3.84 (s, 3H, OCH_3_), 3.61 (s, 3H, N-CH_3_), 2.37 (s, 3H, CH_3_); ^13^C-NMR (DMSO-*d*_6_): δ 188.1, 151.7, 151.3, 150.0, 149.4, 148.0, 147.6, 142.4, 141.7, 137.3, 131.5, 131.3, 129.6, 129.0, 125.9, 125.0, 123.1, 122.7, 121.8, 116.1, 114.8, 111.7, 103.4, 63.0, 56.3, 34.6, 14.1. Anal. calcd for C_27_H_23_Cl_2_N_3_O_4_S: C 58.28; H 4.17; N 7.55. Found: C 58.39; H 4.05; N 7.43.

*1-{4-[(2-Chlorothiazol-5-yl)methoxy]-3-methoxyphenyl}-5-[5-(2-bromophenoxy)-1,3-dimethyl-1H-pyrazol-4-yl]penta-1,4-dien-3-one* (**7j**). Yellow solid, yield 56%, m.p. 165–167 °C. ^1^H-NMR (DMSO-*d*_6_): δ 7.81–7.83 (m, 1H, Ar-H), 7.79 (s, 1H, Thiazole-H), 6.61–7.42 (m, 10H, 2 × CH=CH and Ar-H), 5.37 (s, 2H, CH_2_), 3.84 (s, 3H, OCH_3_), 3.61 (s, 3H, N-CH_3_), 2.37 (s, 3H, CH_3_); ^13^C-NMR (DMSO-*d*_6_): δ 188.1, 152.4, 151.7, 150.0, 149.4, 148.0, 147.6, 142.4, 141.8, 137.3, 134.5, 131.3, 130.3, 129.1, 126.3, 125.1, 123.0, 122.7, 115.9, 114.8, 111.8, 110.9, 103.4, 63.0, 56.3, 34.7, 14.1. Anal. calcd. for C_27_H_23_BrClN_3_O_4_S: C 53.97; H 3.86; N 6.99. Found: C 53.84; H 3.96; N 7.08.

*1-{4-[(2-Chlorothiazol-5-yl)methoxy]-3-methoxyphenyl}-5-[5-(3-bromophenoxy)-1,3-dimethyl-1H-pyrazol-4-yl]penta-1,4-dien-3-one* (**7k**). Yellow oil, yield 58%. ^1^H-NMR (CDCl_3_): δ 7.53 (s, 1H, Thiazole-H), 6.61–7.48 (m, 11H, 2 × CH=CH and Ar-H), 5.25 (s, 2H, CH_2_), 3.91 (s, 3H, OCH_3_), 3.62 (s, 3H, N-CH_3_), 2.42 (s, 3H, CH_3_); ^13^C-NMR (CDCl_3_): δ 188.5, 156.4, 153.0, 150.3, 149.0, 148.6, 147.3, 142.2, 140.5, 136.1, 131.5, 131.4, 129.8, 127.3, 125.3, 123.6, 122.7, 122.4, 118.9, 115.2, 113.8, 111.1, 104.4, 64.0, 56.0, 34.5, 13.9. Anal. calcd. for C_27_H_23_BrClN_3_O_4_S: C 53.97; H 3.86; N 6.99. Found: C 54.09; H 3.73; N 6.87.

*1-{4-[(2-Chlorothiazol-5-yl)methoxy]-3-methoxyphenyl}-5-[5-(2,4-difluorophenoxy)-1,3-dimethyl-1H-pyrazol-4-yl]penta-1,4-dien-3-one* (**7l**). Yellow solid, yield 53%, m.p. 163–165°C. ^1^H-NMR (DMSO-*d*_6_): 7.81 (s, 1H, Thiazole-H), 6.62–7.63 (m, 10H, 2 × CH=CH and Ar-H), 5.37 (s, 2H, CH_2_), 3.83 (s, 3H, OCH_3_), 3.62 (s, 3H, N-CH_3_), 2.37 (s, 3H, CH_3_); ^13^C-NMR (DMSO-*d*_6_): δ 188.0, 151.7, 149.9, 149.4, 147.9, 147.6, 142.5, 141.8, 137.3, 131.3, 129.1, 124.8, 123.4, 123.2, 118.4, 118.3, 114.6, 112.7, 111.8, 106.5, 103.3, 63.0, 56.2, 34.7, 14.4. Anal. calcd. for C_27_H_22_ClF_2_N_3_O_4_S: C 58.12; H 3.97; N 7.53. Found: C 58.01; H 4.09; N 7.64.

*1-{4-[(2-Chlorothiazol-5-yl)methoxy]-3-methoxyphenyl}-5-[5-(3,5-difluorophenoxy)-1,3-dimethyl-1H-pyrazol-4-yl]penta-1,4-dien-3-one* (**7m**). Yellow solid, yield 59%, m.p. 160–162 °C. ^1^H-NMR (DMSO-*d*_6_): 7.80 (s, 1H, Thiazole-H), 6.63–7.47 (m, 10H, 2 × CH=CH and Ar-H), 5.37 (s, 2H, CH_2_), 3.83 (s, 3H, OCH_3_), 3.61 (s, 3H, N-CH_3_), 2.37 (s, 3H, CH_3_); ^13^C-NMR (DMSO-d_6_): δ 188.1, 163.6 (d, *J* = 262 Hz), 157.8, 151.7, 149.9, 149.4, 147.9, 147.0, 142.6, 141.8, 137.3, 131.2, 129.1, 125.0, 123.2, 114.6, 111.8, 103.7, 100.6 (d, *J* = 30 Hz), 63.0, 56.2, 34.7, 14.2. Anal. calcd. for C_27_H_22_ClF_2_N_3_O_4_S: C 58.12; H 3.97; N 7.53. Found: C 58.25; H 3.86; N 7.42.

*1-{4-[(2-Chlorothiazol-5-yl)methoxy]-3-methoxyphenyl}-5-[5-(2,3-dichlorophenoxy)-1,3-dimethyl-1H-pyrazol-4-yl]penta-1,4-dien-3-one* (**7n**). Yellow solid, yield 57%, m.p. 172–174 °C. ^1^H-NMR (DMSO-*d*_6_): 7.81 (s, 1H, Thiazole-H), 6.59–7.51 (m, 10H, 2 × CH=CH and Ar-H), 5.38 (s, 2H, CH_2_), 3.83 (s, 3H, OCH_3_), 3.61 (s, 3H, N-CH_3_), 2.38 (s, 3H, CH_3_); ^13^C-NMR (DMSO-*d*_6_): δ 188.1,152.7, 151.7, 149.9, 149.4, 148.0, 147.1, 142.5, 141.8, 137.9, 137.3, 131.3, 129.8, 129.0, 126.3, 125.0, 123.2, 123.0, 120.9, 114.6, 111.7, 103.5, 63.0, 56.2, 34.7, 14.1. Anal. calcd. for C_27_H_22_Cl_3_N_3_O_4_S: C 54.88; H 3.75; N 7.11. Found: C 54.75; H 3.63; N 7.23.

*1-{4-[(2-Chlorothiazol-5-yl)methoxy]-3-methoxyphenyl}-5-[5-(2,4-dichlorophenoxy)-1,3-dimethyl-1H-pyrazol-4-yl]penta-1,4-dien-3-one* (**7o**). Yellow solid, yield 62%, m.p. 177–179 °C. ^1^H-NMR (DMSO-*d*_6_): 7.90 (d, *J* = 4.0 Hz, 1H, ArH), 7.81 (s, 1H, Thiazole-H), 6.60–7.46 (m, 9H, 2 × CH=CH and Ar-H), 5.38 (s, 2H, CH_2_), 3.84 (s, 3H, OCH_3_), 3.61 (s, 3H, N-CH_3_), 2.38 (s, 3H, CH_3_); ^13^C-NMR (DMSO-*d*_6_): δ 188.0, 151.7, 151.3, 150.5, 149.9, 149.4, 147.9, 147.2, 142.5, 141.8, 137.3, 131.1, 130.9, 129.5, 129.0, 128.9, 124.9, 123.2, 117.4, 114.6, 111.8, 103.5, 63.0, 56.3, 34.7, 14.2. Anal. calcd. for C_27_H_22_Cl_3_N_3_O_4_S: C 54.88; H 3.75; N 7.11. Found: C 55.01; H 3.68; N 7.02.

*1-{4-[(2-Chlorothiazol-5-yl)methoxy]phenyl}-5-[5-(2-chlorophenoxy)-1,3-dimethyl-1H-pyrazol-4-yl]penta-1,4-dien-3-one* (**7p**). Yellow solid, yield 57%, m.p. 189–191 °C. ^1^H-NMR (CDCl_3_): 7.58 (s, 1H, Thiazole-H), 6.66–7.56 (m, 12H, 2 × CH=CH and Ar-H), 5.23 (s, 2H, CH_2_), 3.67 (s, 3H, N-CH_3_), 2.43 (s, 3H, CH_3_); ^13^C-NMR (DMSO-*d*_6_): δ 188.1, 159.7, 151.7, 151.3, 148.0, 147.5, 142.0, 141.8, 137.2, 132.1, 131.5, 131.4, 130.8, 129.7, 128.4, 125.9, 124.4, 123.0, 121.8, 116.1, 115.9, 115.0, 103.4, 62.3, 34.6, 14.1. Anal. calcd. for C_26_H_21_Cl_2_N_3_O_3_S: C 59.32; H 4.02; N 7.98. Found: C 59.45; H 4.13; N 7.87.

*1-{4-[(2-Chlorothiazol-5-yl)methoxy]phenyl}-5-[5-(2-bromophenoxy)-1,3-dimethyl-1H-pyrazol-4-yl]penta-1,4-dien-3-one* (**7q**). Yellow oil, yield 61%. ^1^H-NMR (CDCl_3_): 6.63–7.72 (m, 13H, Thiazole-H, 2 × CH=CH and Ar-H), 5.23 (s, 2H, CH_2_), 3.67 (s, 3H, N-CH_3_), 2.42 (s, 3H, CH_3_); ^13^C-NMR (DMSO-*d*_6_): δ 188.0, 159.7, 152.3, 151.7, 148.0, 147.6, 142.0, 141.8, 137.2, 134.5, 131.4, 130.8, 130.3, 128.4, 126.3, 124.5, 122.9, 115.9, 115.8, 111.0, 103.4, 62.3, 34.7, 14.1. Anal. calcd. for C_26_H_21_BrClN_3_O_3_S: C 54.70; H 3.71; N 7.36. Found: C 54.58; H 3.81; N 7.47.

*1-{4-[(2-Chloropyridin-5-yl)methoxy]phenyl}-5-[5-(2-trifluoromethoxyphenoxy)-1,3-dimethyl-1H-pyrazol-4-yl]penta-1,4-dien-3-one* (**7r**). Yellow solid, yield 58%, m.p. 185–186 °C. ^1^H-NMR (DMSO-*d*_6_): 8.53 (S, 1H, Py-H), 6.56–7.97 (m, 14H, 2 × CH=CH, Py-H and Ar-H), 5.22 (s, 2H, CH_2_), 3.57 (s, 3H, N-CH_3_), 2.39 (s, 3H, CH_3_); ^13^C-NMR (DMSO-*d*_6_): δ 188.0, 160.3, 150.4, 149.7, 147.8, 146.9, 142.0, 139.9, 137.0, 132.4, 131.1, 130.8, 130.7, 130.0, 128.2, 125.5, 124.7, 124.6, 124.2, 123.4, 123.2 (q, *J* = 230 Hz), 116.2, 115.8, 103.7, 66.7, 34.6, 14.3. Anal. calcd. for C_29_H_23_ClF_3_N_3_O_4_: C 61.11; H 4.07; N 7.37. Found: C 61.02; H 4.20; N 7.25.

*1-{4-[(2-Chloropyridin-5-yl)methoxy]phenyl}-5-[5-(3-methylphenoxy)-1,3-dimethyl-1H-pyrazol-4-yl]penta-1,4-dien-3-one* (**7s**). Yellow solid, yield 63%, m.p. > 280 °C. ^1^H-NMR (DMSO-*d*_6_): 8.54 (d, *J* = 2.0 Hz, 1H, Py-H), 6.61–7.98 (m, 14H, 2 × CH=CH, Py-H and Ar-H), 5.23 (s, 2H, CH_2_), 3.57 (s, 3H, N-CH_3_), 2.37 (s, 3H, CH_3_), 2.30 (s, 3H, Ar-CH_3_); ^13^C-NMR (DMSO-*d*_6_): δ 188.1, 160.3, 156.2, 150.4, 149.8, 148.2, 147.7, 142.5, 142.0, 140.8, 139.9, 132.5, 131.7, 130.8, 130.6, 128.4, 128.3, 125.2, 124.7, 124.4, 123.1, 116.2, 115.8, 112.6, 103.7, 66.7, 34.6, 21.4, 14.6. Anal. calcd. for C_29_H_26_ClN_3_O_3_: C 69.66; H 5.24; N 8.40. Found: C 69.75; H 5.11; N 8.28.

*1-{4-[(2-Chloropyridin-5-yl)methoxy]phenyl}-5-[5-(2-chlorophenoxy)-1,3-dimethyl-1H-pyrazol-4-yl]penta-1,4-dien-3-one* (**7t**). Yellow solid, yield 55%, m.p. 205–207 °C. ^1^H-NMR (DMSO-*d*_6_): 8.55 (d, *J* = 2.4 Hz, 1H, Py-H), 6.58–7.98 (m, 14H, 2 × CH=CH, Py-H and Ar-H), 5.23 (s, 2H, CH_2_), 3.61 (s, 3H, N-CH_3_), 2.37 (s, 3H, CH_3_); ^13^C-NMR (DMSO-*d*_6_): δ 188.1, 160.3, 151.4, 150.4, 149.8, 147.9, 147.6, 142.0, 139.9, 132.5, 131.5, 131.3, 130.8, 130.7, 129.6, 128.4, 128.2, 125.9, 124.7, 124.4, 123.0, 121.9, 116.2, 115.8, 103.4, 66.7, 34.7, 14.1. Anal. calcd. for C_28_H_23_Cl_2_N_3_O_3_: C 64.62; H 4.45; N 8.07. Found: C 64.74; H 4.57; N 8.01.

*1-{4-[(2-Chloropyridin-5-yl)methoxy]phenyl}-5-[5-(3-chlorophenoxy)-1,3-dimethyl-1H-pyrazol-4-yl]penta-1,4-dien-3-one* (**7u**). Yellow solid, yield 63%, m.p. 179–181 °C. ^1^H-NMR (DMSO-*d*_6_): 6.60–8.54 (m, 15H, 2 × CH=CH, Py-H and Ar-H), 5.22 (s, 2H, CH_2_), 3.59 (s, 3H, N-CH_3_), 2.37 (s, 3H, CH_3_); ^13^C-NMR (DMSO-*d*_6_): δ 188.1, 160.3, 156.8, 150.4, 149.8, 147.9, 147.4, 142.1, 139.9, 134.9, 132.4, 132.3, 131.4, 130.8, 128.2, 124.7, 124.6, 124.3, 123.4, 116.3, 115.7, 114.4, 103.7, 66.7, 34.7, 14.1. Anal. calcd. for C_28_H_23_Cl_2_N_3_O_3_: C 64.62; H 4.45; N 8.07. Found: C 64.49; H 4.55; N 8.19.

*1-{4-[(2-Chloropyridin-5-yl)methoxy]phenyl}-5-[5-(2-bromophenoxy)-1,3-dimethyl-1H-pyrazol-4-yl]penta-1,4-dien-3-one* (**7v**). Yellow solid, yield 62%, m.p. 202–204 °C. ^1^H-NMR (DMSO-*d*_6_): 8.54 (s, 1H, Py-H), 6.58–7.98 (m, 14H, 2 × CH=CH, Py-H and Ar-H), 5.23 (s, 2H, CH_2_), 3.61 (s, 3H, N-CH_3_), 2.37 (s, 3H, CH_3_); ^13^C-NMR (DMSO-*d*_6_): δ 188.0, 160.3, 152.4, 150.4, 149.8, 148.0, 147.6, 142.1, 139.9, 134.5, 132.5, 130.8, 130.3, 128.1, 126.3, 124.7, 124.4, 115.9, 115.8, 111.0, 103.4, 66.7, 34.7, 14.1. Anal. calcd. for C_28_H_23_BrClN_3_O_3_: C 59.54; H 4.10; N 7.44. Found: C 59.66; H 4.01; N 7.32.

*1-{4-[(2-Chloropyridin-5-yl)methoxy]phenyl}-5-[5-(3-bromophenoxy)-1,3-dimethyl-1H-pyrazol-4-yl]penta-1,4-dien-3-one* (**7w**). Yellow solid, yield 67%, m.p. 157–159 °C. ^1^H-NMR (DMSO-*d*_6_): 8.53 (s,1H, Py-H), 6.60–7.97 (m, 14H, 2 × CH=CH, Py-H and Ar-H), 5.22 (s, 2H, CH_2_), 3.59 (s, 3H, N-CH_3_), 2.37 (s, 3H, CH_3_); ^13^C-NMR (DMSO-*d*_6_): δ 188.0, 159.8, 156.3, 149.9, 149.3, 147.4, 147.0, 141.7, 139.4, 132.2, 132.0, 130.9, 130.4, 127.8, 127.1, 124.3, 123.8, 122.9, 122.7, 118.6, 115.3, 114.3, 103.2, 66.2, 34.2, 13.7. Anal. calcd. for C_28_H_23_BrClN_3_O_3_: C 59.54; H 4.10; N 7.44. Found: C 59.43; H 4.23; N 7.56.

*1-{4-[(2-Chloropyridin-5-yl)methoxy]phenyl}-5-[5-(2,3-difluorophenoxy)-1,3-dimethyl-1H-pyrazol-4-yl]penta-1,4-dien-3-one* (**7x**). Yellow solid, yield 52%, m.p. 205–207 °C. ^1^H-NMR (DMSO-*d*_6_): 8.54 (s, 1H, Py-H), 6.61–7.98 (m, 13H, 2 × CH=CH, Py-H and Ar-H), 5.23 (s, 2H, CH_2_), 3.63 (s, 3H, N-CH_3_), 2.38 (s, 3H, CH_3_). ^13^C-NMR (DMSO-*d*_6_): δ 188.0, 160.3, 150.4, 149.8, 147.8, 147.1, 142.2, 139.9, 132.4, 131.1, 130.8, 130.7, 128.2, 125.6, 125.5, 125.4, 124.7, 124.2, 123.7, 115.8, 113.3, 113.2, 112.5, 103.6, 66.7, 34.7, 14.2. Anal. calcd. for C_28_H_22_ClF_2_N_3_O_3_: C 64.43; H 4.25; N 8.05. Found: C 64.30; H 4.37; N 8.15.

*1-{4-[(2-Chloropyridin-5-yl)methoxy]phenyl}-5-[5-(2,3-dichlorophenoxy)-1,3-dimethyl-1H-pyrazol-4-yl]penta-1,4-dien-3-one* (**7y**). Yellow solid, yield 58%, m.p. 139–141 °C. ^1^H-NMR (DMSO-*d*_6_): 8.54 (s,1H, Py-H), 6.56–7.98 (m, 13H, 2 × CH=CH, Py-H and Ar-H), 5.23 (s, 2H, CH_2_), 3.61 (s, 3H, N-CH_3_), 2.38 (s, 3H, CH_3_). ^13^C-NMR (DMSO-*d*_6_): δ 188.0, 160.3, 152.7, 150.4, 149.8, 148.0, 147.1, 142.2, 139.9, 133.8, 132.4, 130.8, 129.8, 128.1, 126.3, 124.7, 124.3, 115.8, 114.6, 103.5, 66.7, 34.7, 14.1. Anal. calcd. for C_28_H_22_Cl_3_N_3_O_3_: C 60.61; H 4.00; N 7.57. Found: C 60.73; H 3.89; N 7.48.

*1-{4-[(2-Chloropyridin-5-yl)methoxy]phenyl}-5-[5-(2,4-dichlorophenoxy)-1,3-dimethyl-1H-pyrazol-4-yl]penta-1,4-dien-3-one* (**7z**). Yellow solid, yield 60%, m.p. 174–176 °C. ^1^H-NMR (DMSO-*d*_6_): 8.54 (d, *J* = 2.4 Hz, 1H, Py-H), 6.57–7.98 (m, 13H, 2 × CH=CH, Py-H and Ar-H), 5.23 (s, 2H, CH_2_), 3.60 (s, 3H, N-CH_3_), 2.37 (s, 3H, CH_3_). ^13^C-NMR (DMSO-*d*_6_): δ 188.0, 160.3, 150.4, 150.3, 149.8, 148.0, 147.1, 142.2, 139.9, 132.4, 131.1, 130.9, 130.8, 129.5, 129.0, 128.2, 124.7, 124.2, 123.3, 123.0, 117.4, 115.8, 103.5, 66.7, 34.7, 14.1. Anal. calcd. for C_28_H_22_Cl_3_N_3_O_3_: C 60.61; H 4.00; N 7.57. Found: C 60.50; H 4.13; N 7.69.

### 3.2. Anticancer Activity Assay

Antiproliferative activities of the newly synthesized compounds were evaluated in vitro against SGC-7901 (human gastric cancer cells), HepG2 (human hepatoma cells), and Panc-1 (human pancreatic carcinoma cells). Different cancer cells were plated in a 96-well flat-bottom tissue culture plate at a density of 10^4^ cells/mL in Dulbecco’s modified Eagle’s medium (DMEM) and 10% fetal bovine serum and allowed to adhere overnight at 37 °C in 5% CO_2_ [33]. The cells were incubated in triplicate with, or without, different concentrations of each test compound for 48 h. During the last 4 h incubation, 30 μL of tetrazolium dye (MTT) solution (5 mg/mL) was added to each well. The resulting MTT-formazan crystals were dissolved in 150 μL DMSO, and absorbance was measured spectrophotometrically at 570 nm using an ELISA plate reader. The inhibition induced by each test compound at the indicated concentrations was expressed as a percentage. The concentration required for 50% inhibition (IC_50_) was calculated using the software (Graph Pad Prism, San Diego, CA, USA, Version 4.03).

## 4. Conclusions

In conclusion, a number of novel penta-1,4-dien-3-one derivatives bearing a substituted pyrazole moiety were synthesized and tested for their anticancer activities. Preliminary bioassays showed that some of the target compounds exhibited more potent antiproliferative activity against HepG2 cells than SGC-7901 and Panc-1 cells. The IC_50_ values of **7a**–**m**, **o**, **r**, **s**, **u**, **w**, **y** and **z** against HepG2 cell lines were 0.10–5.05 μM, respectively, better than that of the control sorafenib (IC_50_ = 16.20 μM). To obtain more active derivatives, further studies on these compounds are well under way.

## Figures and Tables

**Table 1:** Antiproliferative activity of compounds **7a**–**7z**.

Compounds	IC_50_, μM		
	SGC-7901	HepG2	Panc-1
**7a**	>40	3.23 ± 0.35	>40
**7b**	>40	1.60 ± 0.22	>40
**7c**	>40	1.90 ± 0.32	>40
**7d**	>40	1.68 ± 0.26	>40
**7e**	>40	2.02 ± 0.29	>40
**7f**	>40	2.60 ± 0.25	>40
**7g**	>40	1.44 ± 0.36	>40
**7h**	>40	5.05 ± 0.52	>40
**7i**	>40	3.89 ± 0.38	>40
**7j**	>40	4.63 ± 0.45	>40
**7k**	>40	4.59 ± 0.33	>40
**7l**	>40	1.86 ± 0.27	>40
**7m**	>40	2.03 ± 0.39	>40
**7n**	>40	>40	>40
**7o**	>40	4.71 ± 0.56	>40
**7p**	>40	>40	>40
**7q**	>40	>40	>40
**7r**	>40	0.76 ± 0.20	>40
**7s**	>40	0.25 ± 0.05	>40
**7t**	>40	>40	>40
**7u**	>40	0.15 ± 0.02	>40
**7v**	>40	>40	>40
**7w**	>40	0.18 ± 0.04	>40
**7x**	>40	>40	>40
**7y**	>40	0.28 ± 0.02	>40
**7z**	>40	0.10 ± 0.03	>40
Sorafenib	12.10 ± 2.68	16.20 ± 2.17	11.50 ± 2.32

## Supplementary Materials

Supplementary materials (^1^H-NMR and ^13^C-NMR spectra of pyrazole oxime compounds **7a**–**7z**) are available online.
