# Supplementary material for: Design, Synthesis and Bioactivities of Novel 1,4-Pentadien-3-one Derivatives Containing a Substituted Pyrazolyl Moiety
Source: Molecules. 2017 Jul 6;22(7):1126. doi: 10.3390/molecules22071126 (PMC6152210; doi:10.3390/molecules22071126)
Supplement: Supplementary file 1 [file molecules-22-01126-s001.pdf]

molecules

MDPI

Supplementary Materials

The ^{1}H-NMR and ^{13}C-NMR spectra of pyrazole oxime derivatives (**7a–7z**) were listed below:

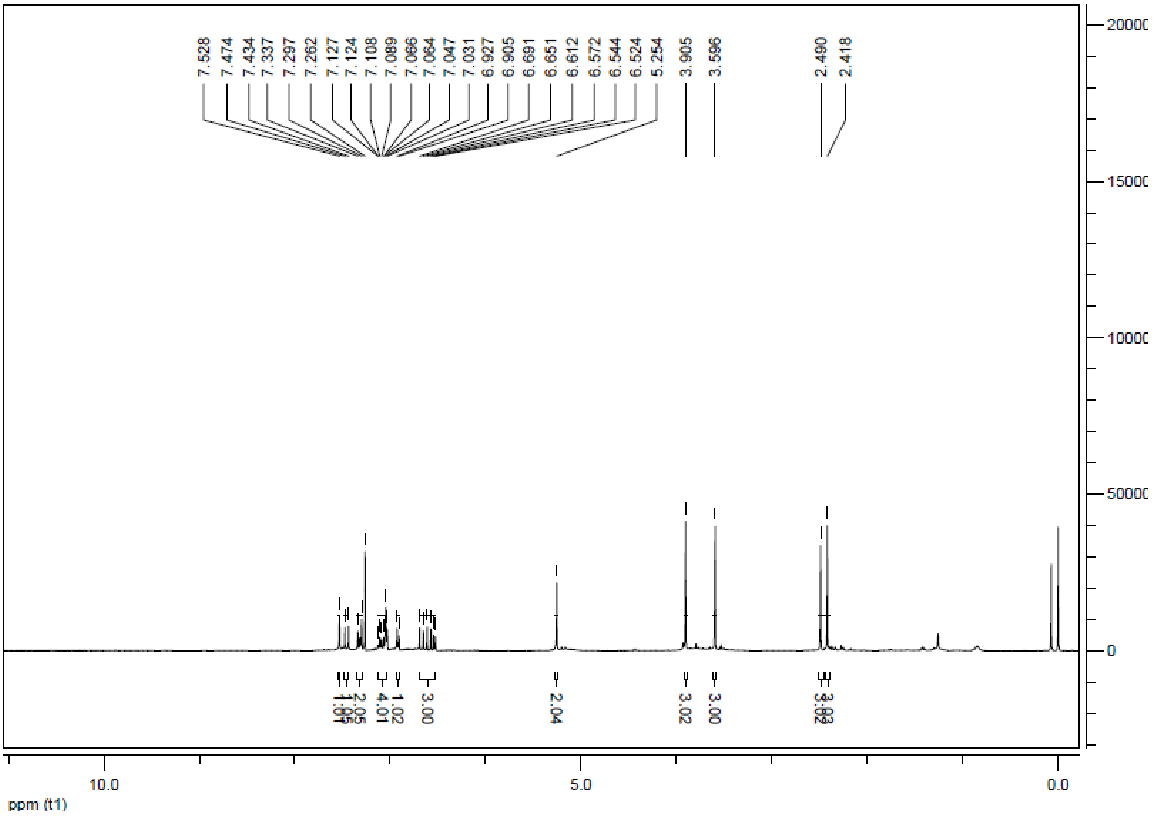

Figure S1. ^{1}H-NMR of compound **7a** (400 MHz, $CDCl_3$).

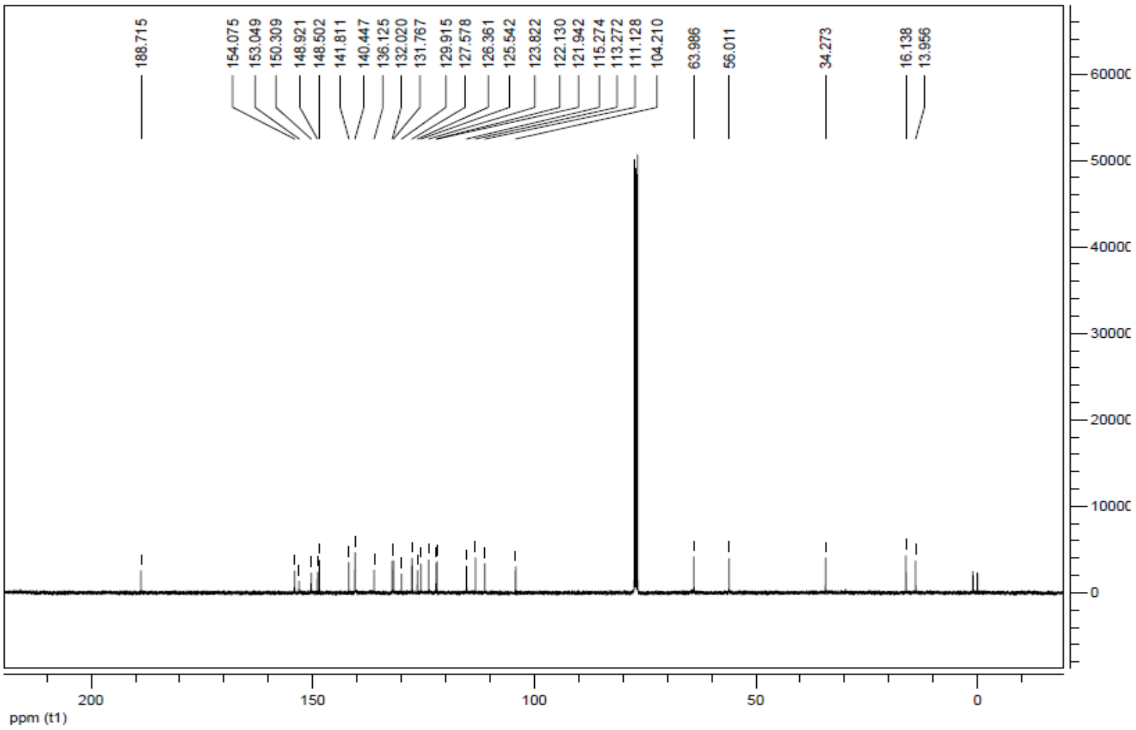

Figure S2. ^{13}C-NMR of compound **7a** (100 MHz, $CDCl_3$).

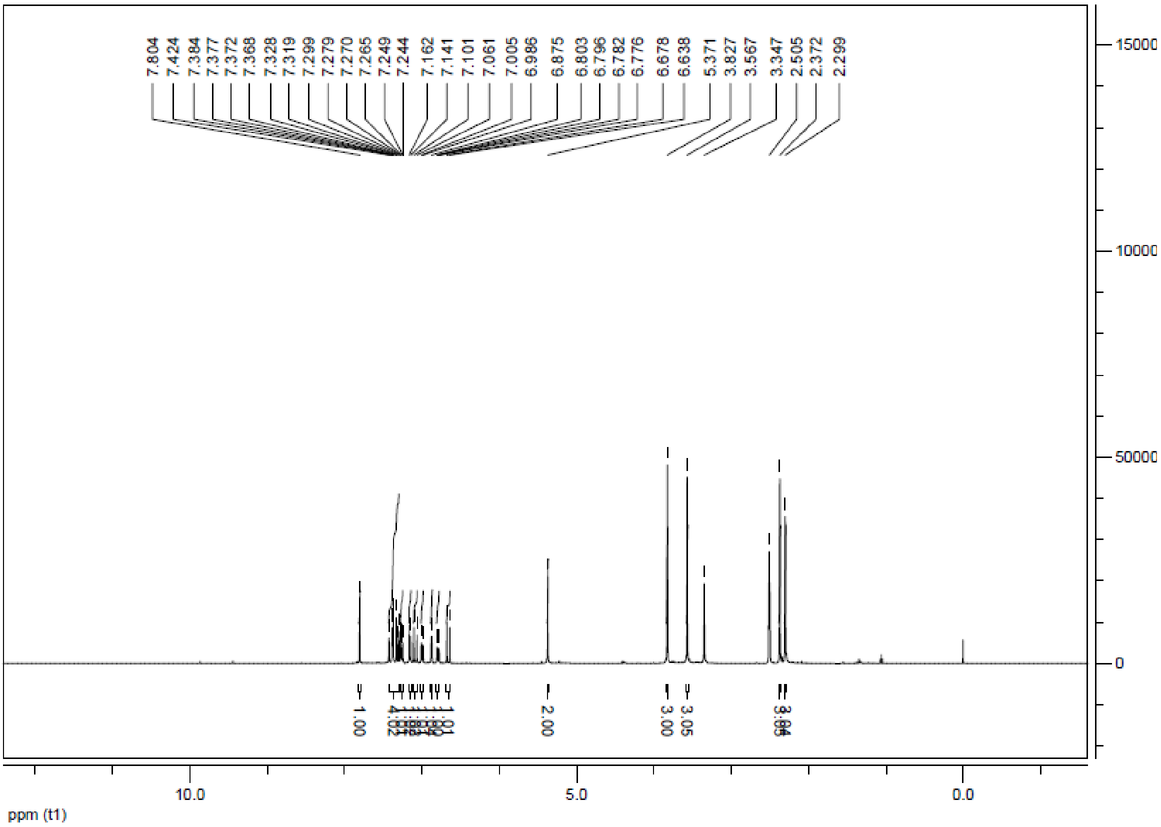

Figure S3. ¹H-NMR of compound **7b** (400 MHz, DMSO-*d*₆).

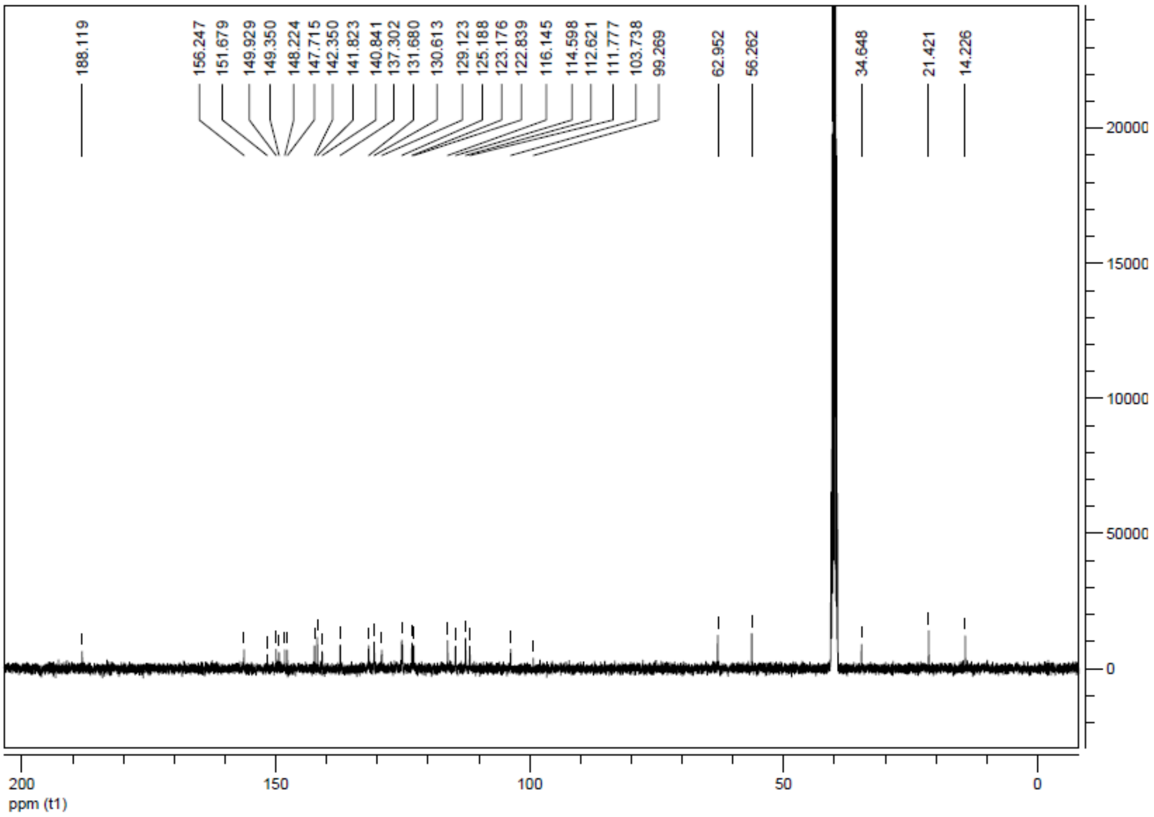

Figure S4. ¹³C-NMR of compound **7b** (100 MHz, DMSO-*d*₆).

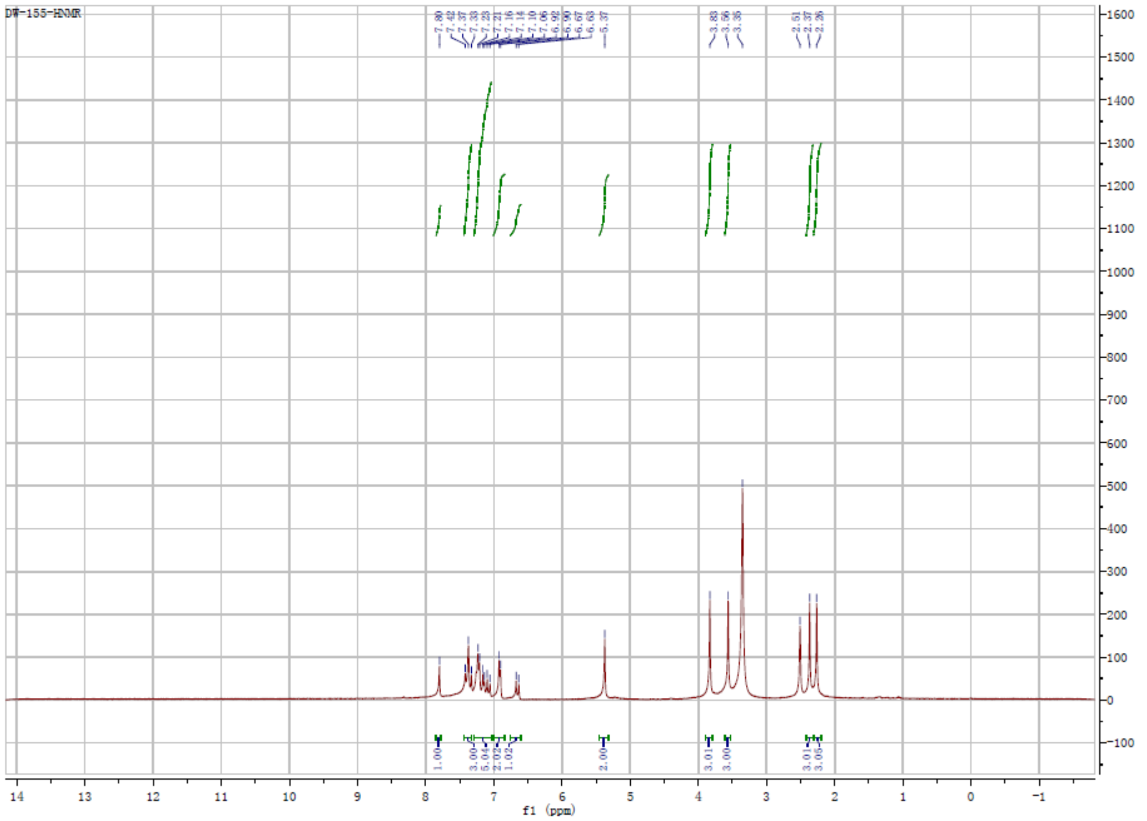

Figure S5. ^{1}H-NMR of compound **7c** (400 MHz, DMSO-d_6).

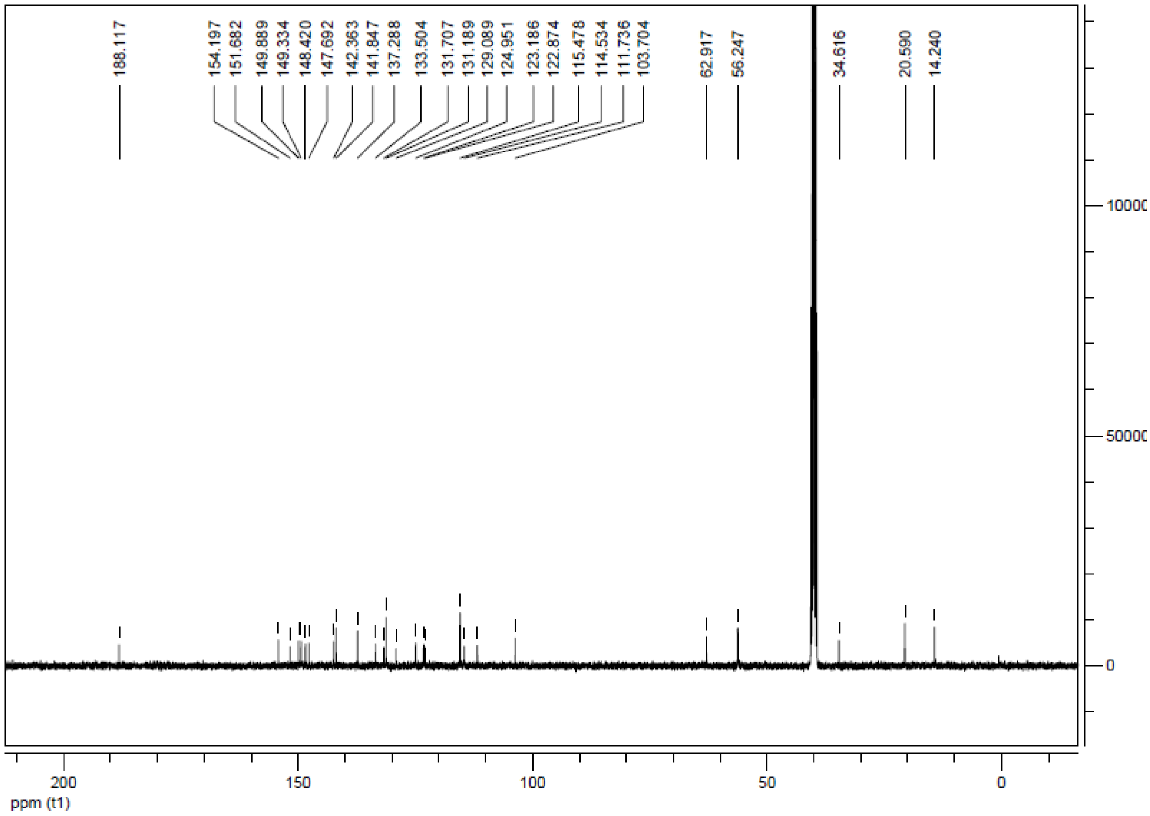

Figure S6. ^{13}C-NMR of compound **7c** (100 MHz, DMSO-d_6).

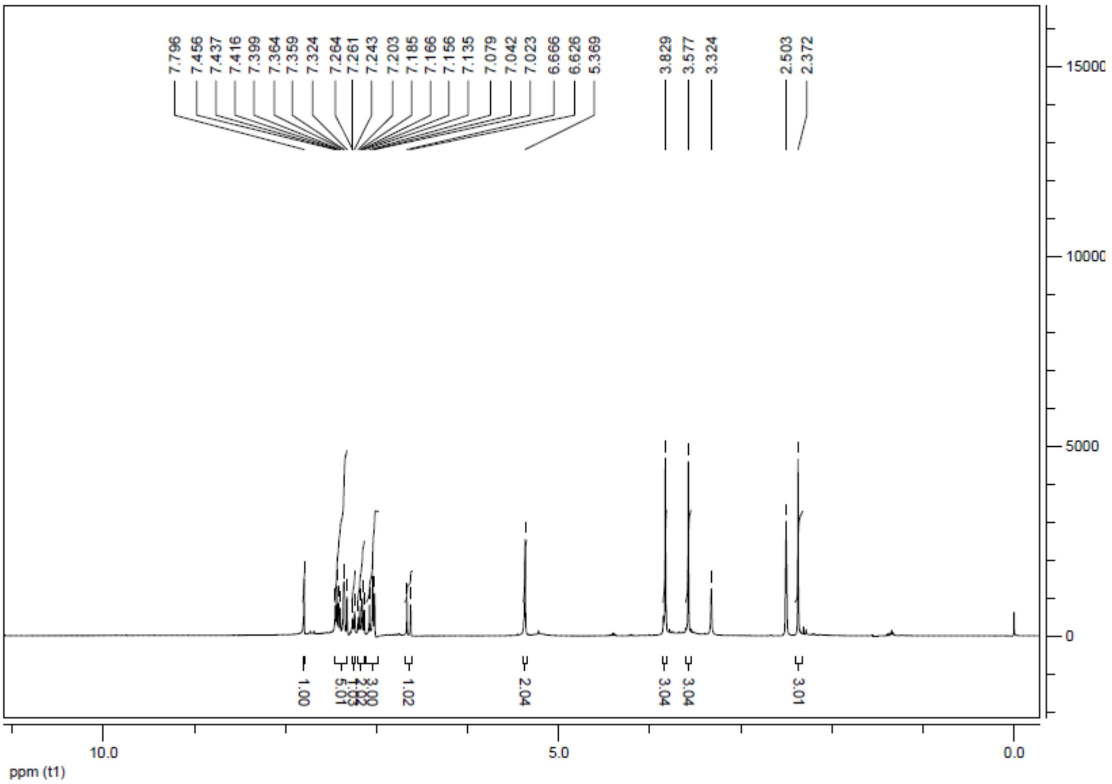

Figure S7. ^{1}H-NMR of compound **7d** (400 MHz, DMSO-d_6).

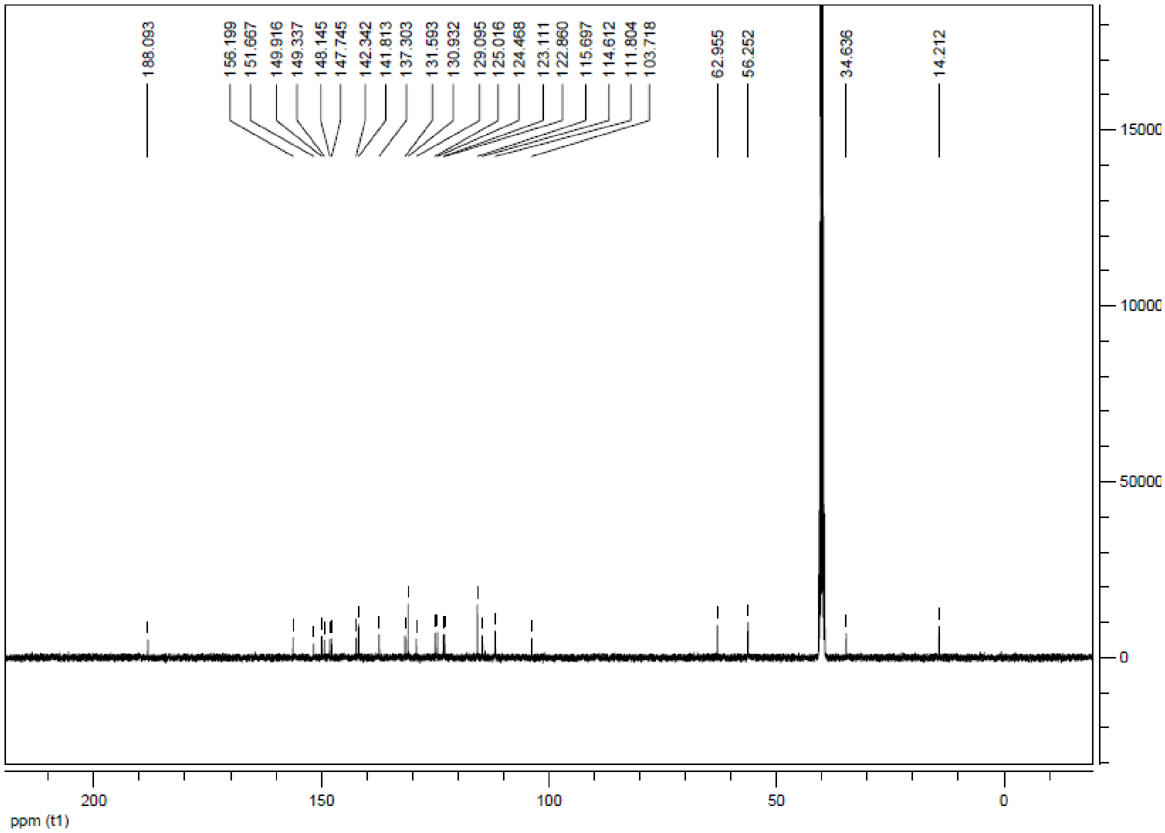

Figure S8. ^{13}C-NMR of compound **7d** (100 MHz, DMSO-d_6).

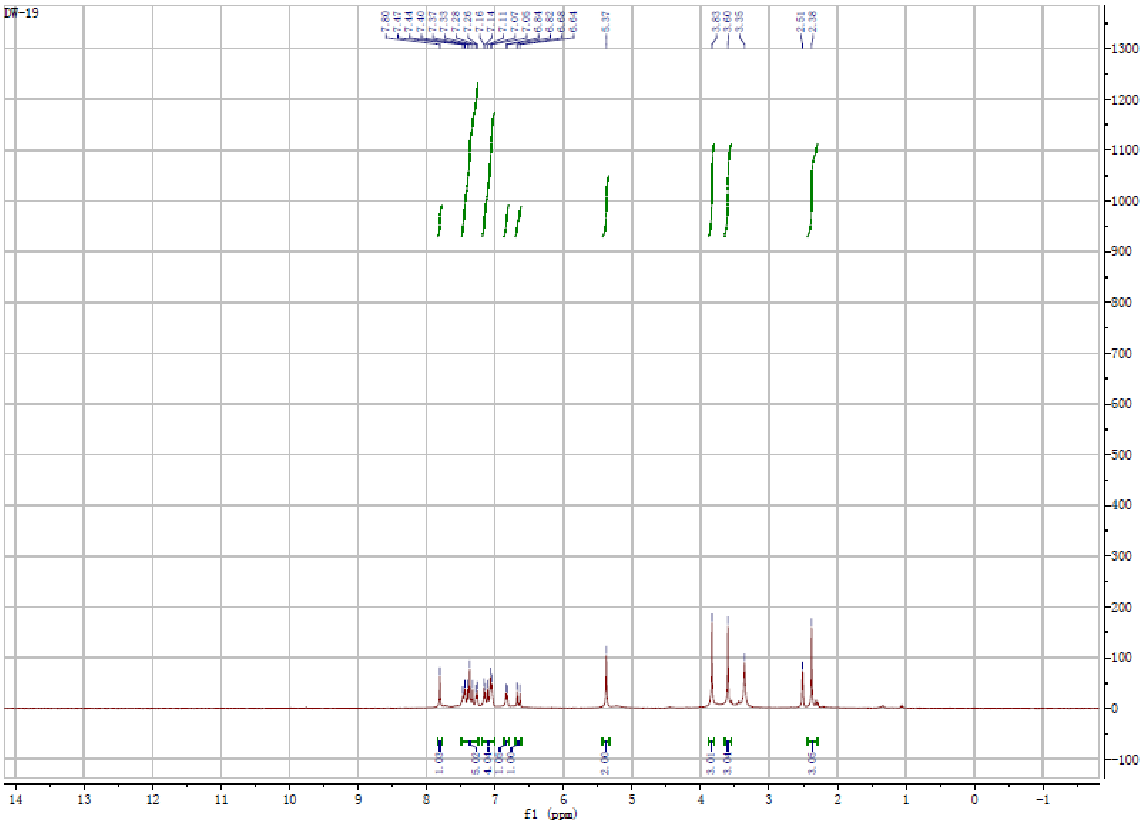

Figure S9. ^{1}H-NMR of compound **7e** (400 MHz, DMSO-*d*6).

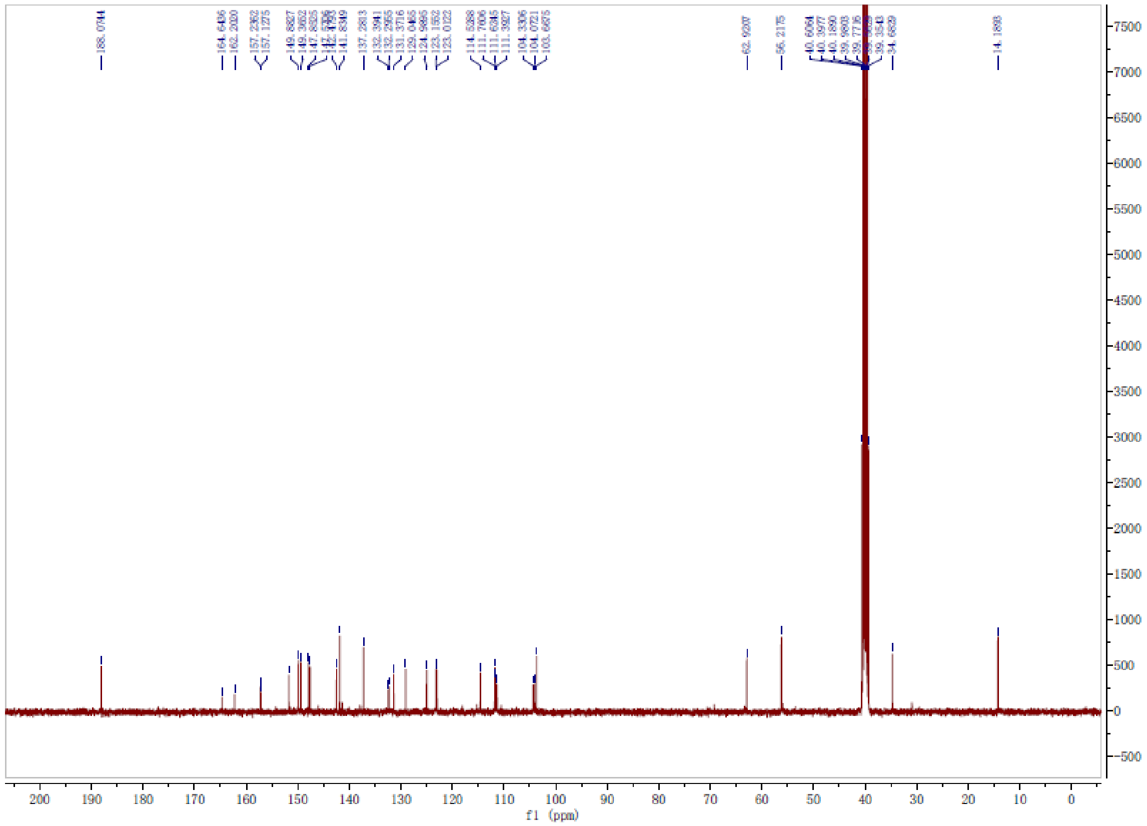

Figure S10. ^{13}C-NMR of compound **7e** (100 MHz, DMSO-*d*6).

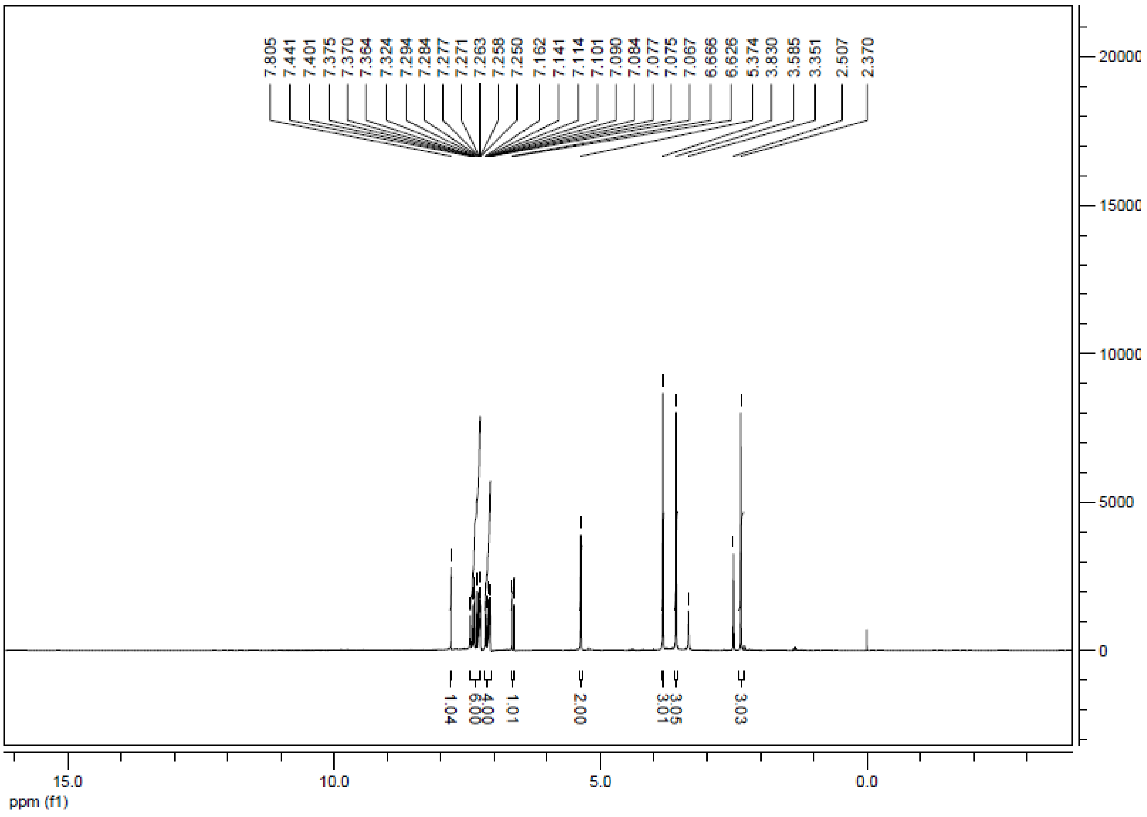

Figure S11. ^{1}H-NMR of compound **7f** (400 MHz, DMSO-*d*6).

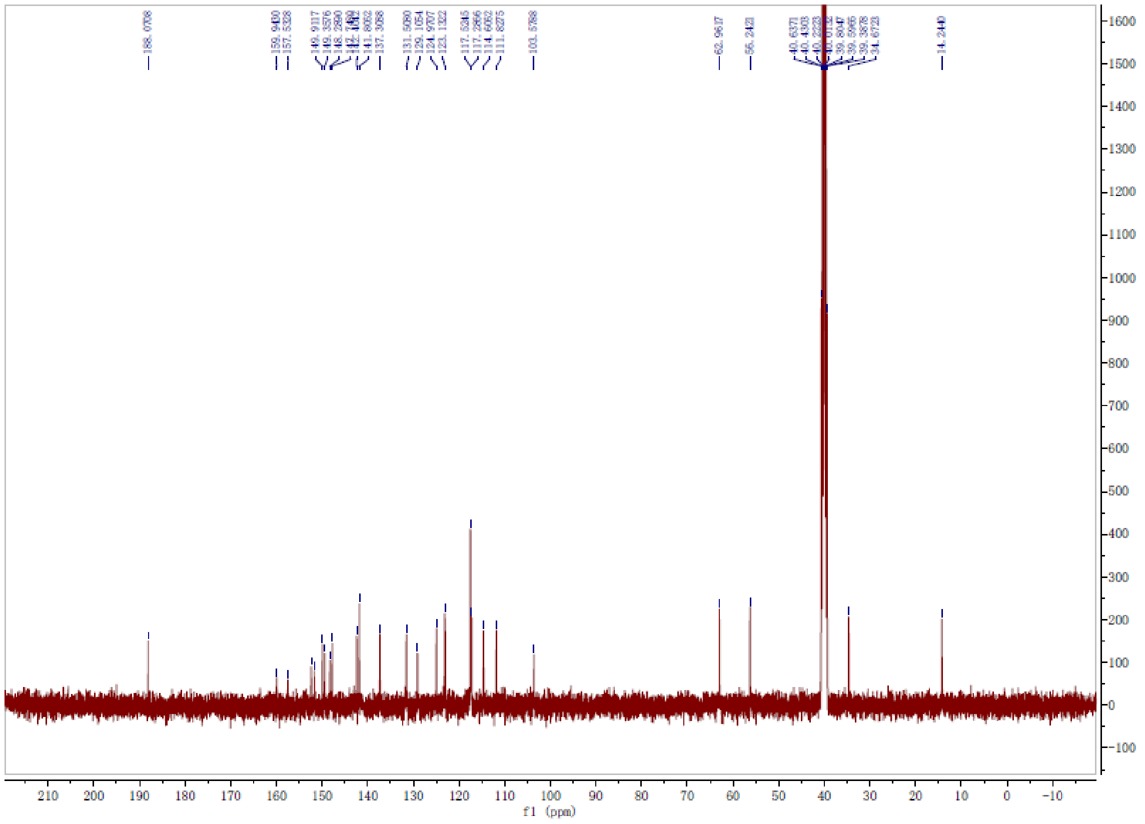

Figure S12. ^{13}C-NMR of compound **7f** (100 MHz, DMSO-*d*6).

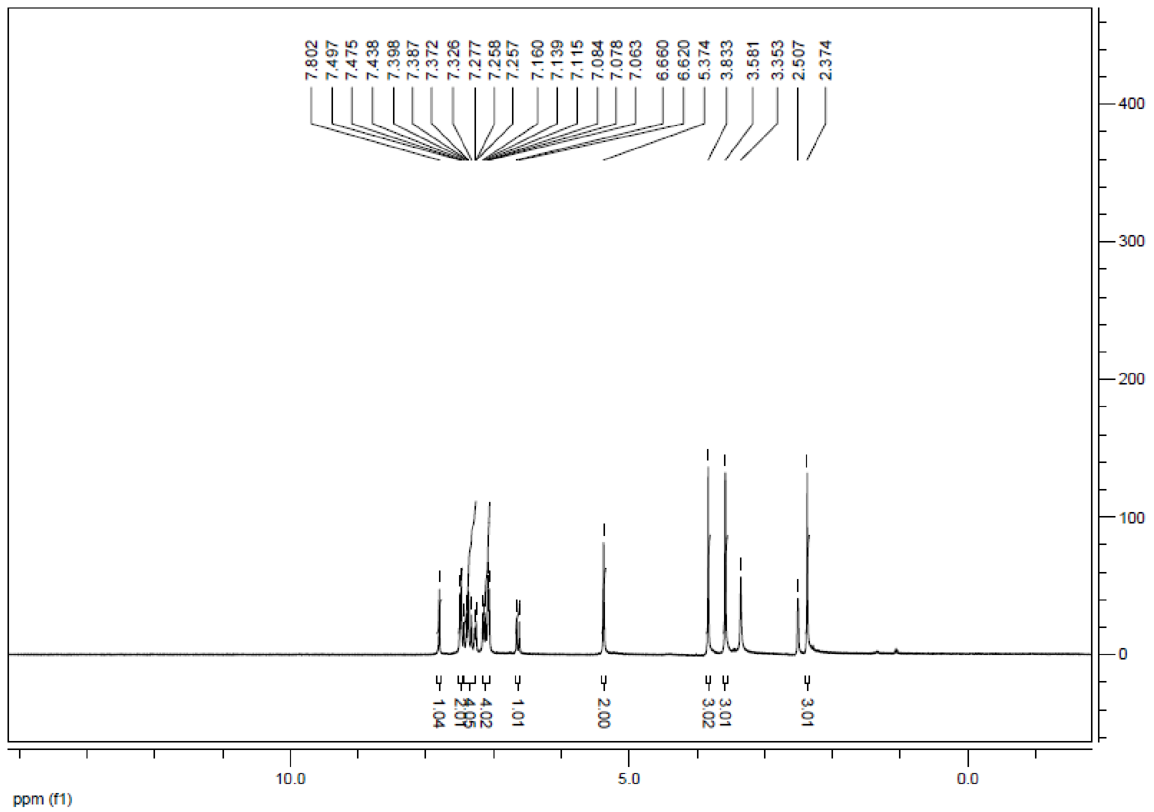

Figure S13. ^{1}H-NMR of compound **7g** (400 MHz, DMSO-*d*6).

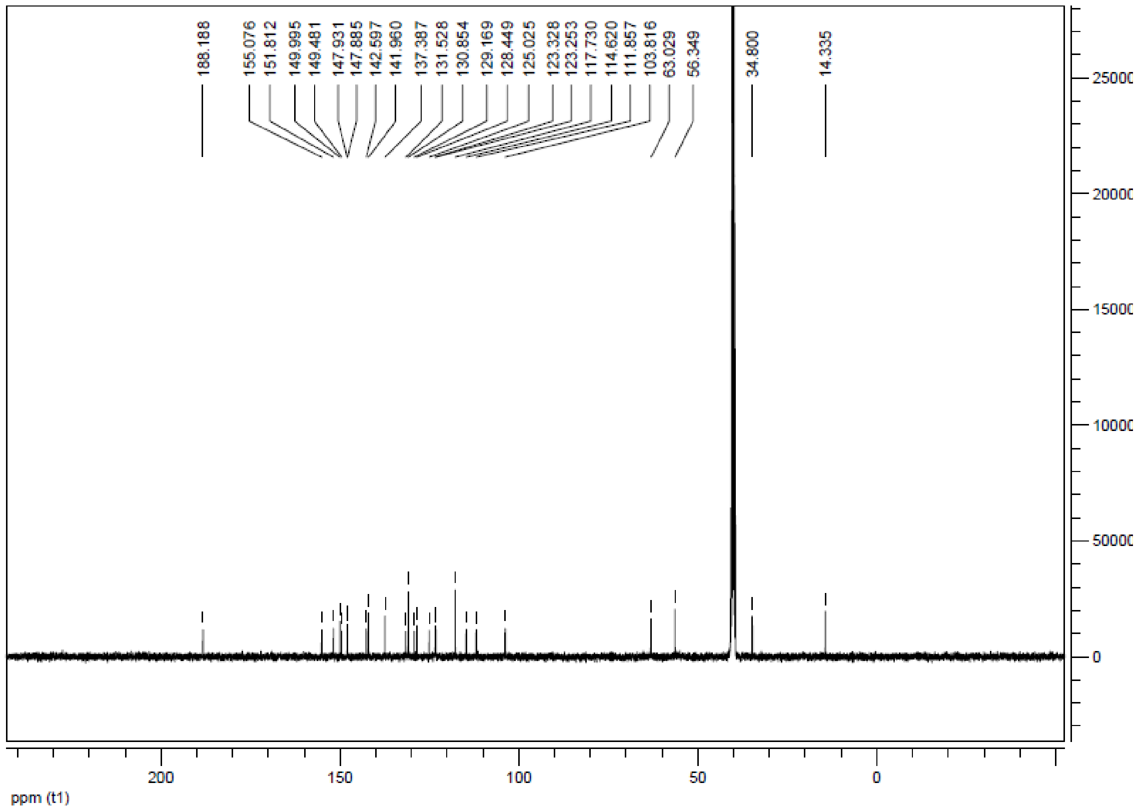

Figure S14. ^{13}C-NMR of compound **7g** (100 MHz, DMSO-*d*6).

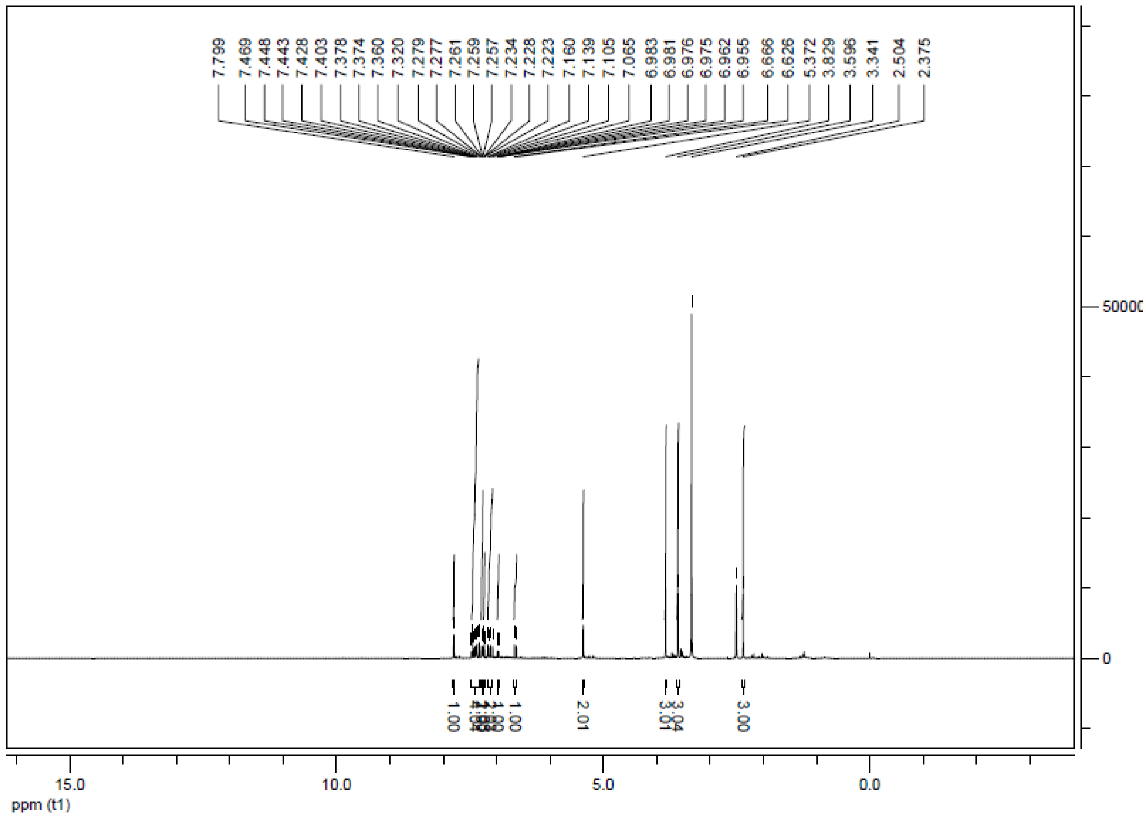

Figure S15. ^{1}H-NMR of compound **7h** (400 MHz, DMSO-d_6).

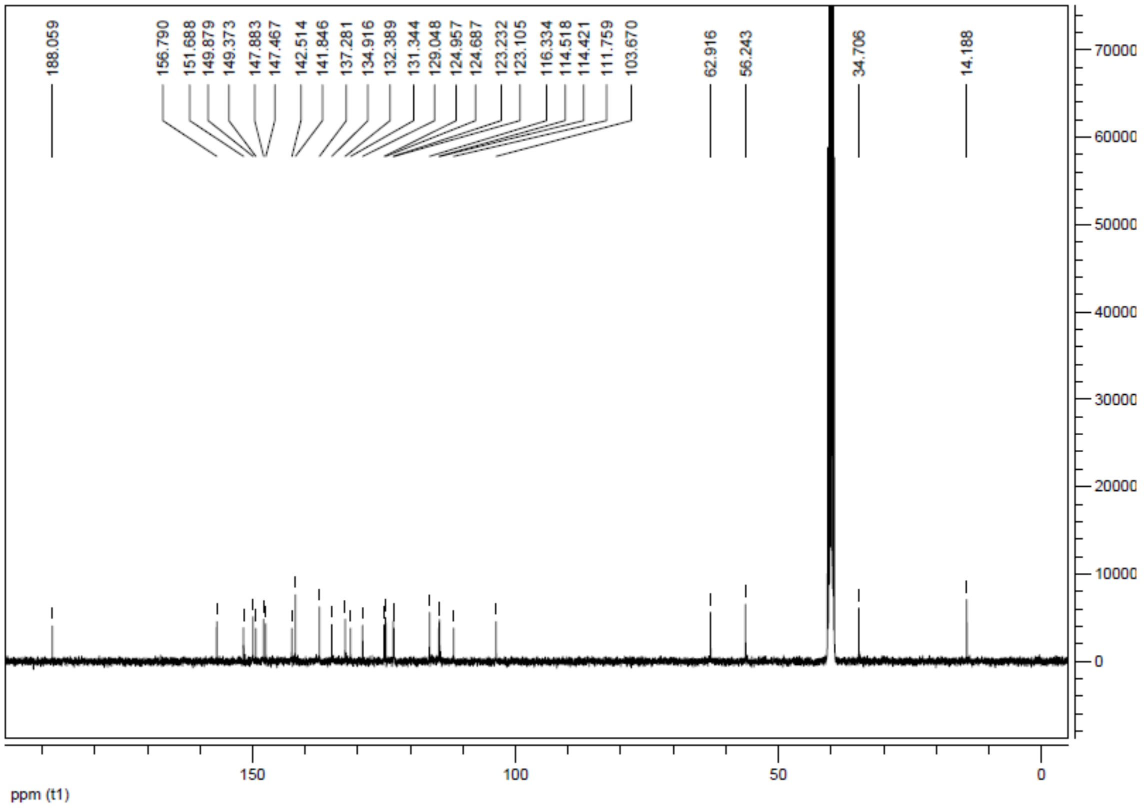

Figure S16. ^{13}C-NMR of compound **7h** (100 MHz, DMSO-d_6).

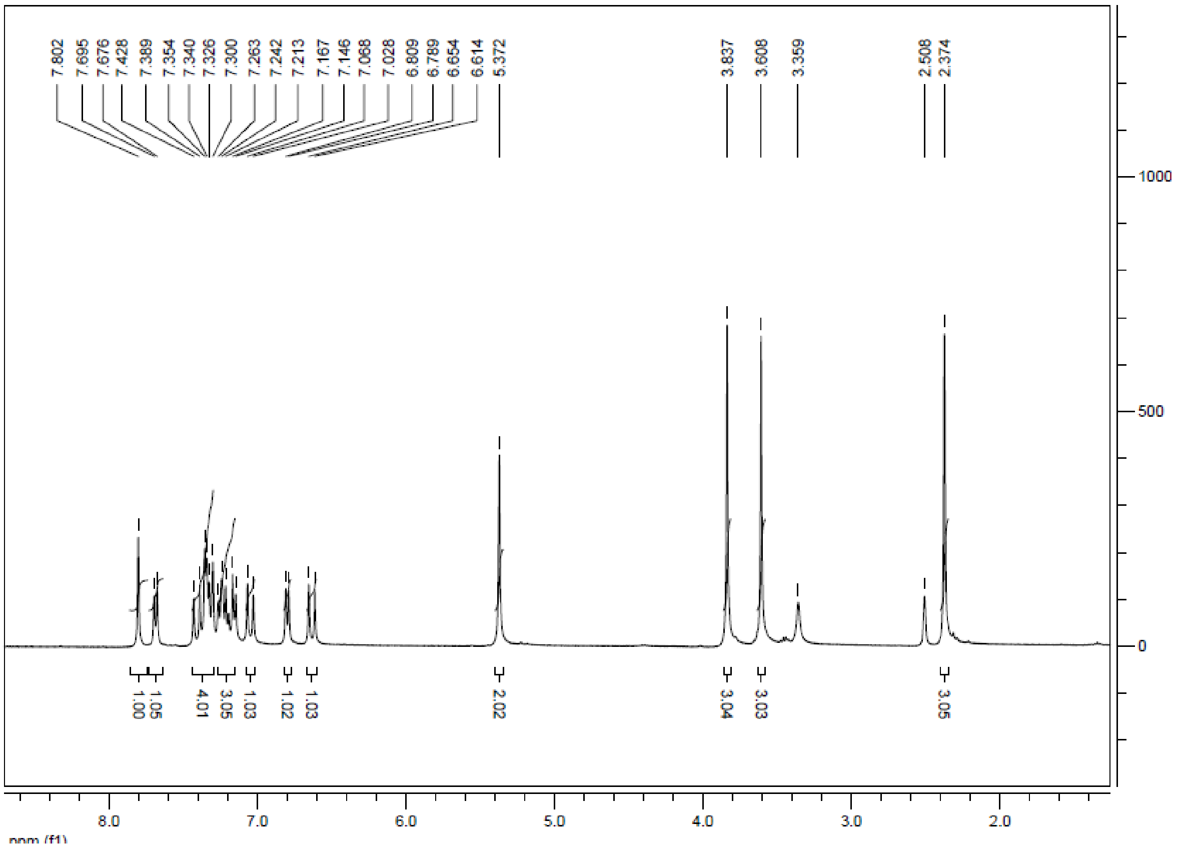

Figure S17. ^{1}H-NMR of compound **9i** (400 MHz, DMSO-d_6).

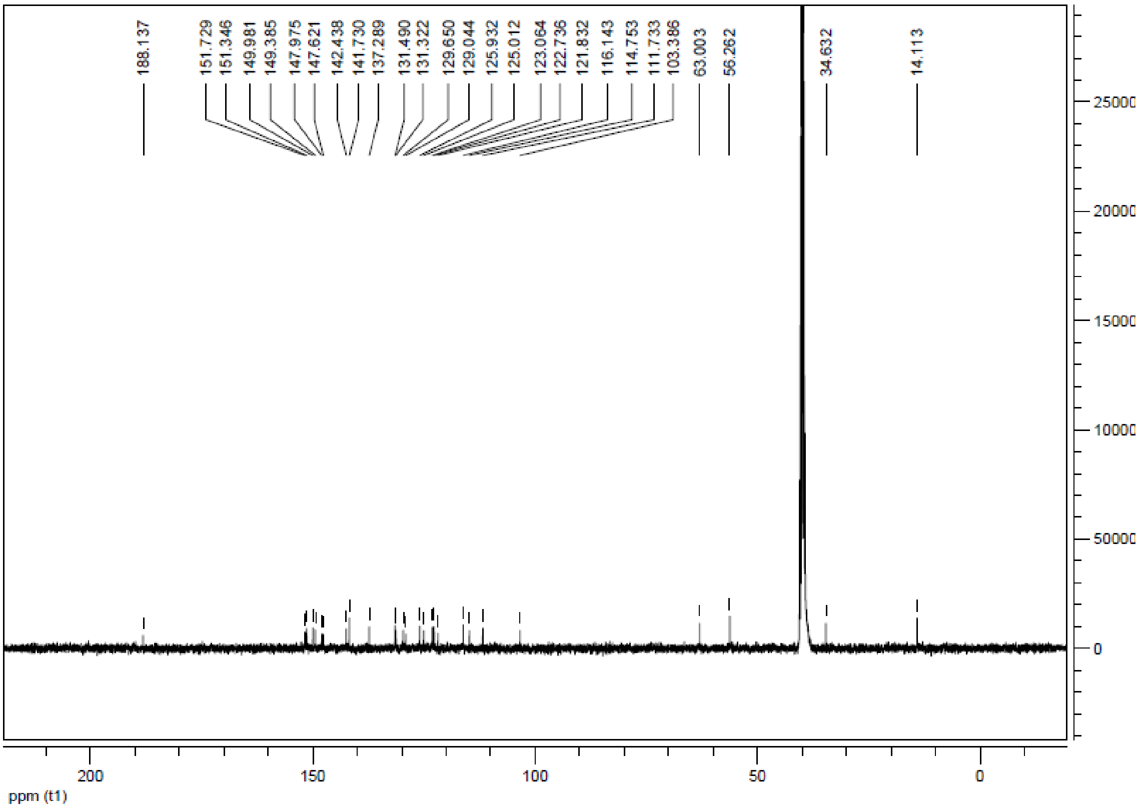

Figure S18. ^{13}C-NMR of compound **7i** (100 MHz, DMSO-d_6).

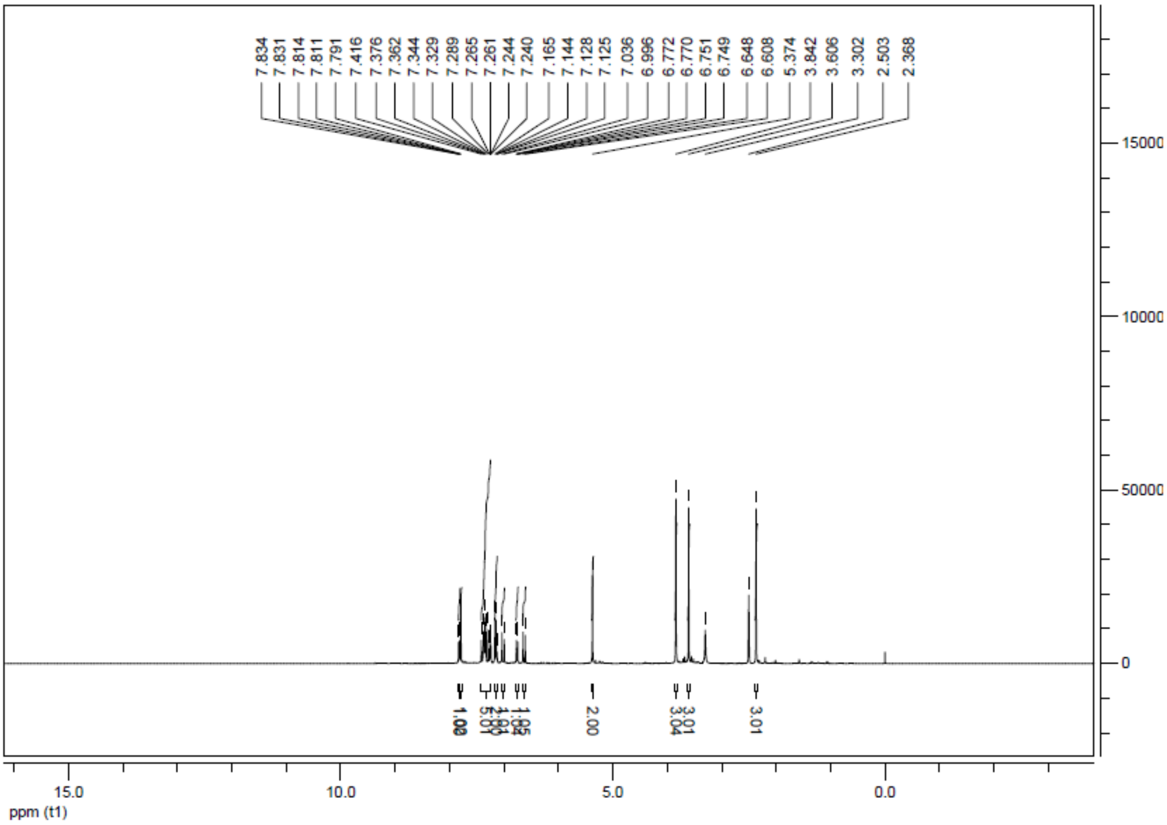

Figure S19. ^{1}H-NMR of compound **7j** (400 MHz, DMSO-*d*6).

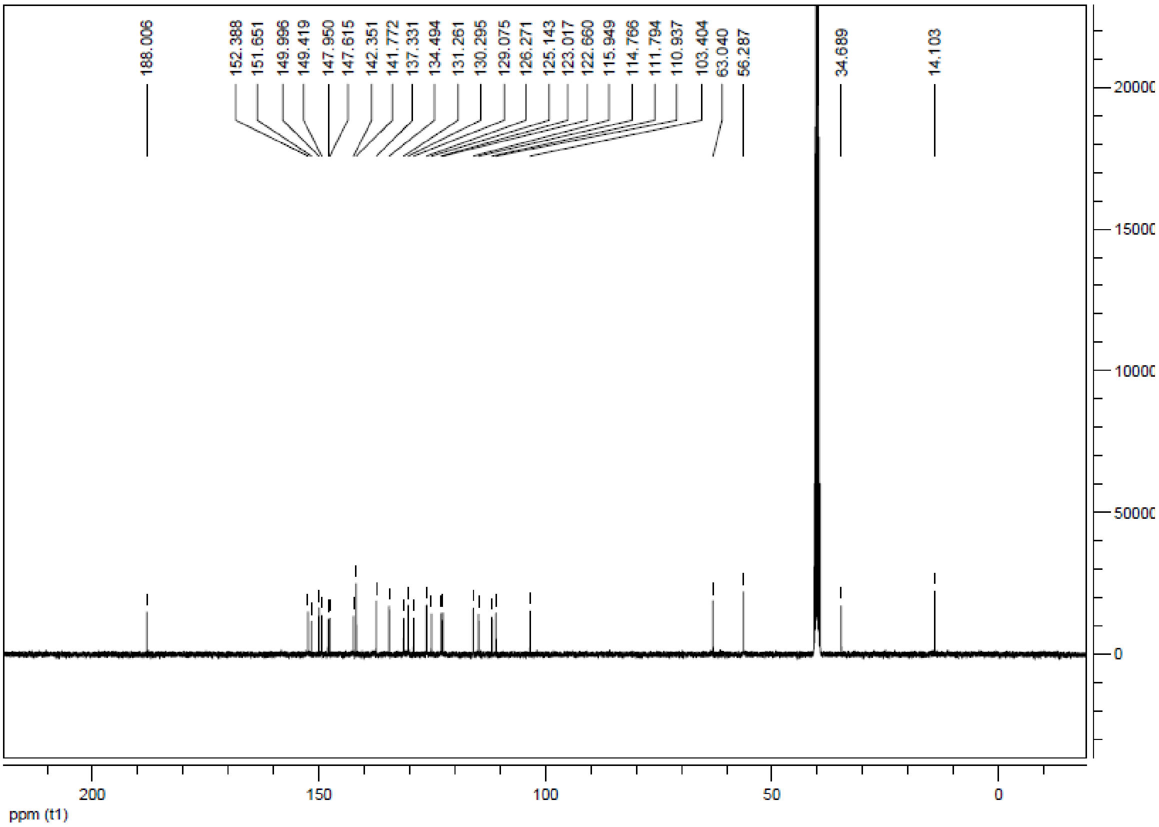

Figure S20. ^{13}C-NMR of compound **7j** (100 MHz, DMSO-*d*6).

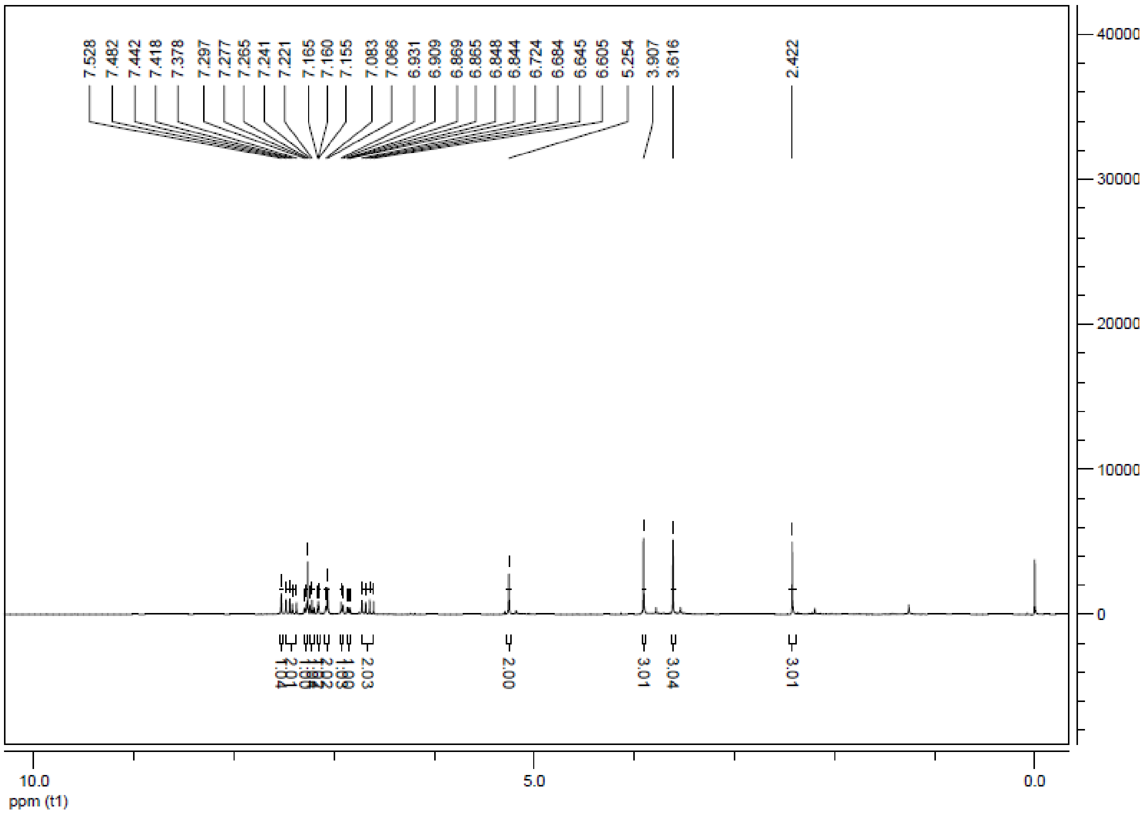

Figure S21. ^{1}H-NMR of compound **7k** (400 MHz, $CDCl_3$).

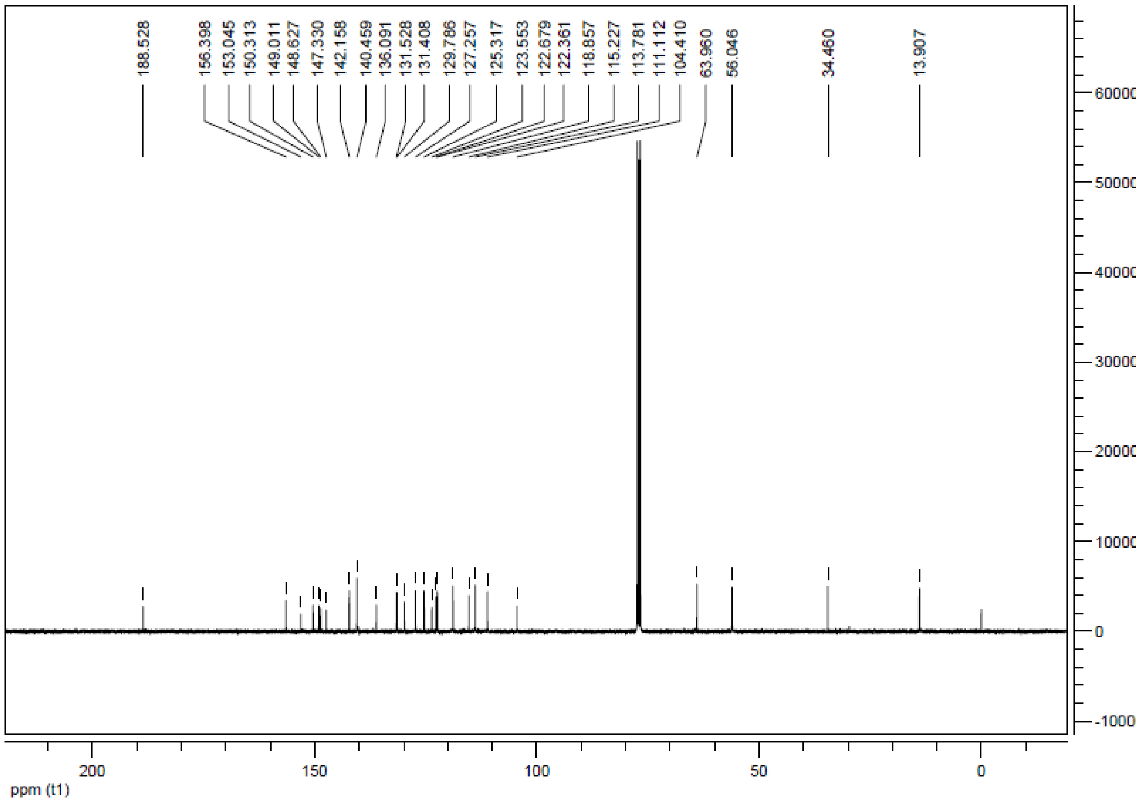

Figure S22. ^{13}C-NMR of compound **7k** (100 MHz, $CDCl_3$).

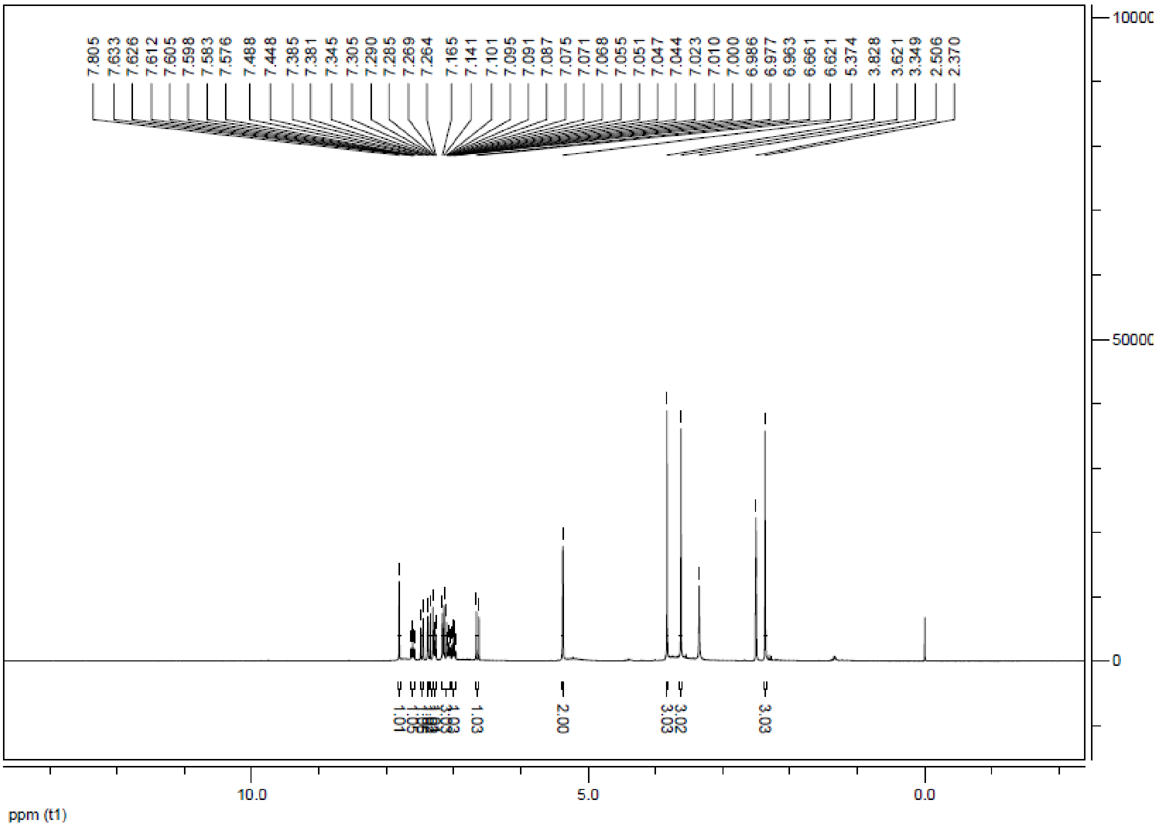

Figure S23. ^{1}H-NMR of compound **7l** (400 MHz, DMSO-*d*$_6$).

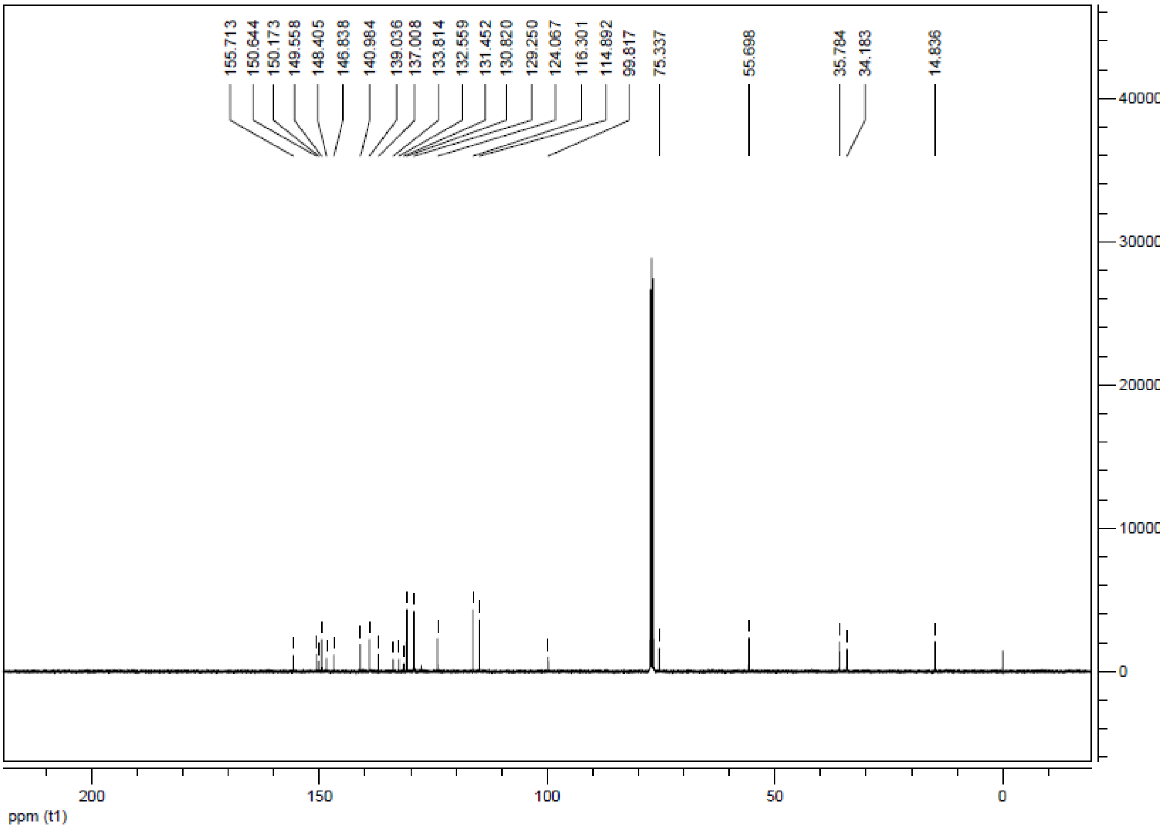

Figure S24. ^{13}C-NMR of compound **7l** (100 MHz, DMSO-*d*$_6$).

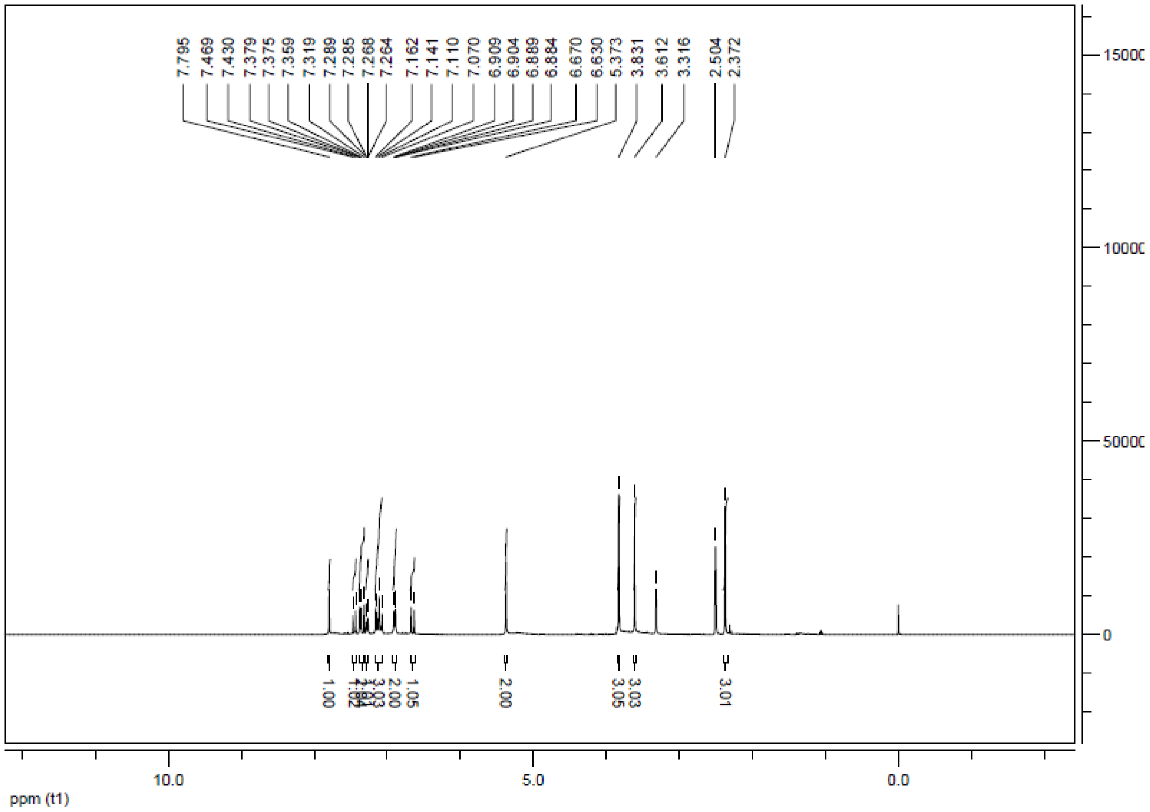

Figure S25. ¹H-NMR of compound **7m** (400 MHz, DMSO-d_6).

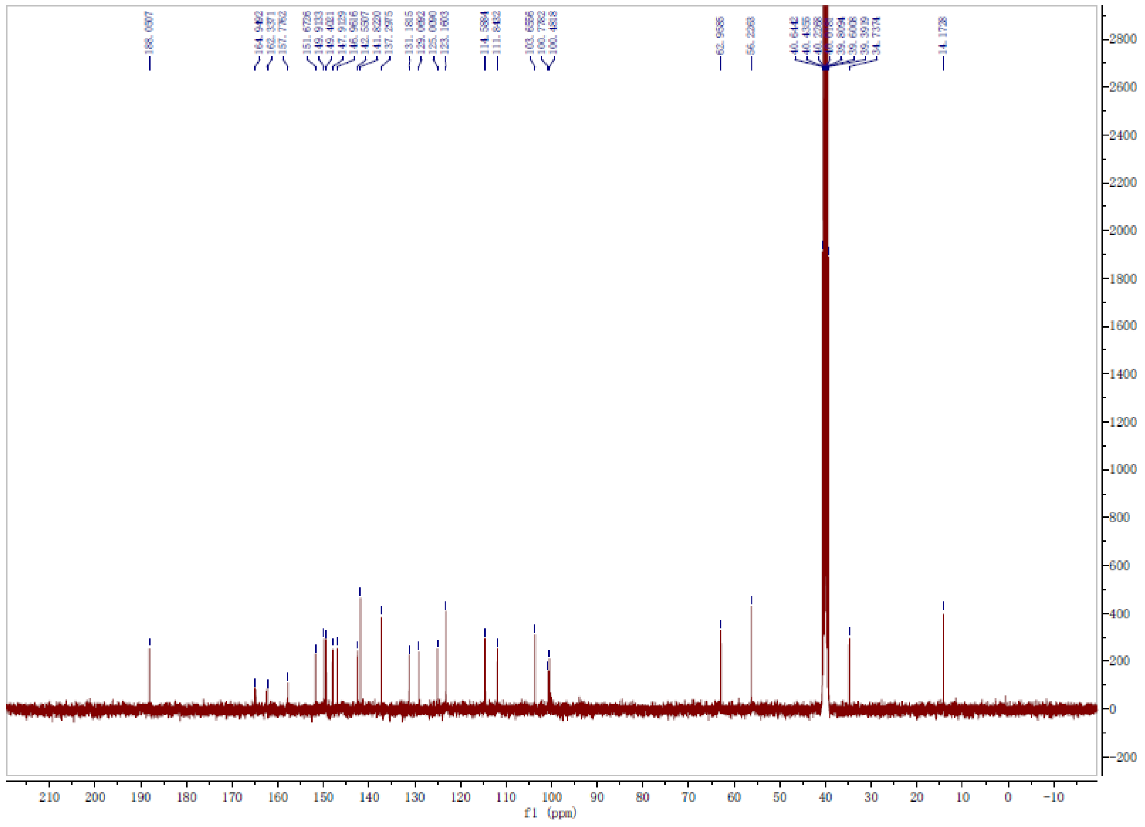

Figure S26. ¹³C-NMR of compound **7m** (100 MHz, DMSO-d_6).

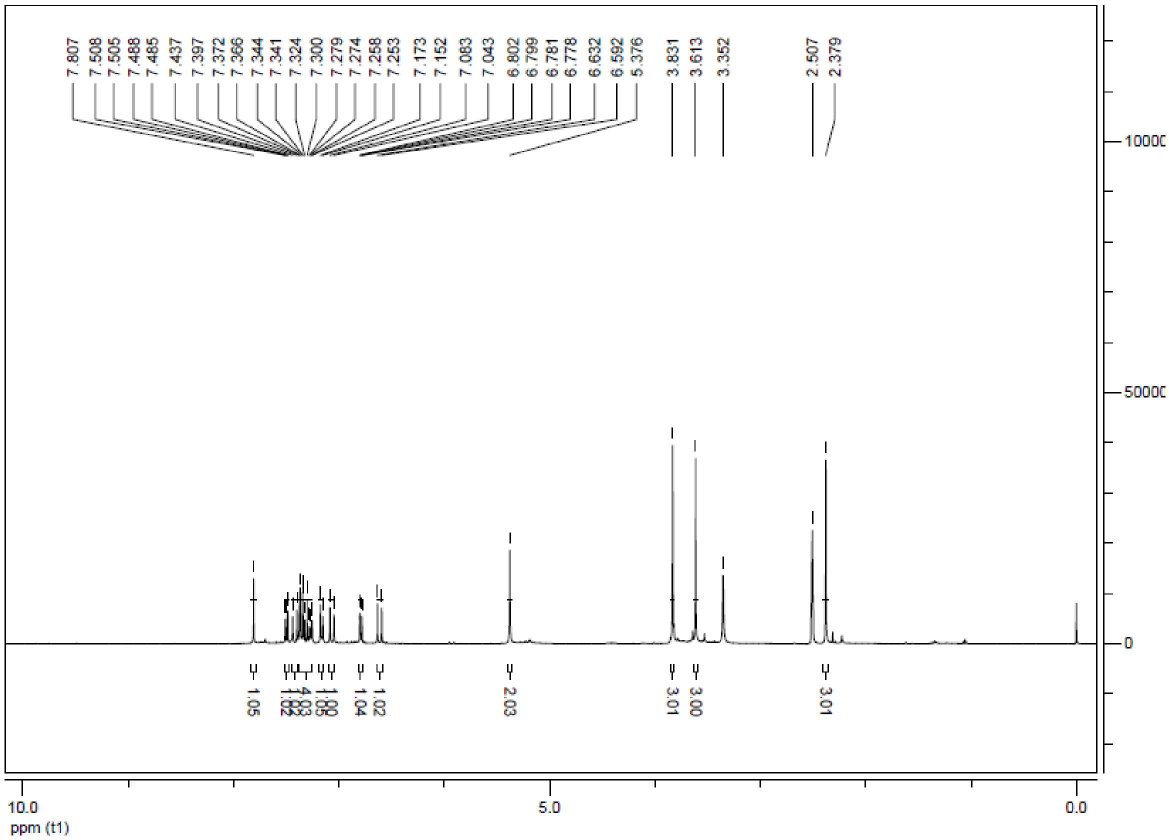

Figure S27. ^{1}H-NMR of compound **7n** (400 MHz, DMSO-*d*6).

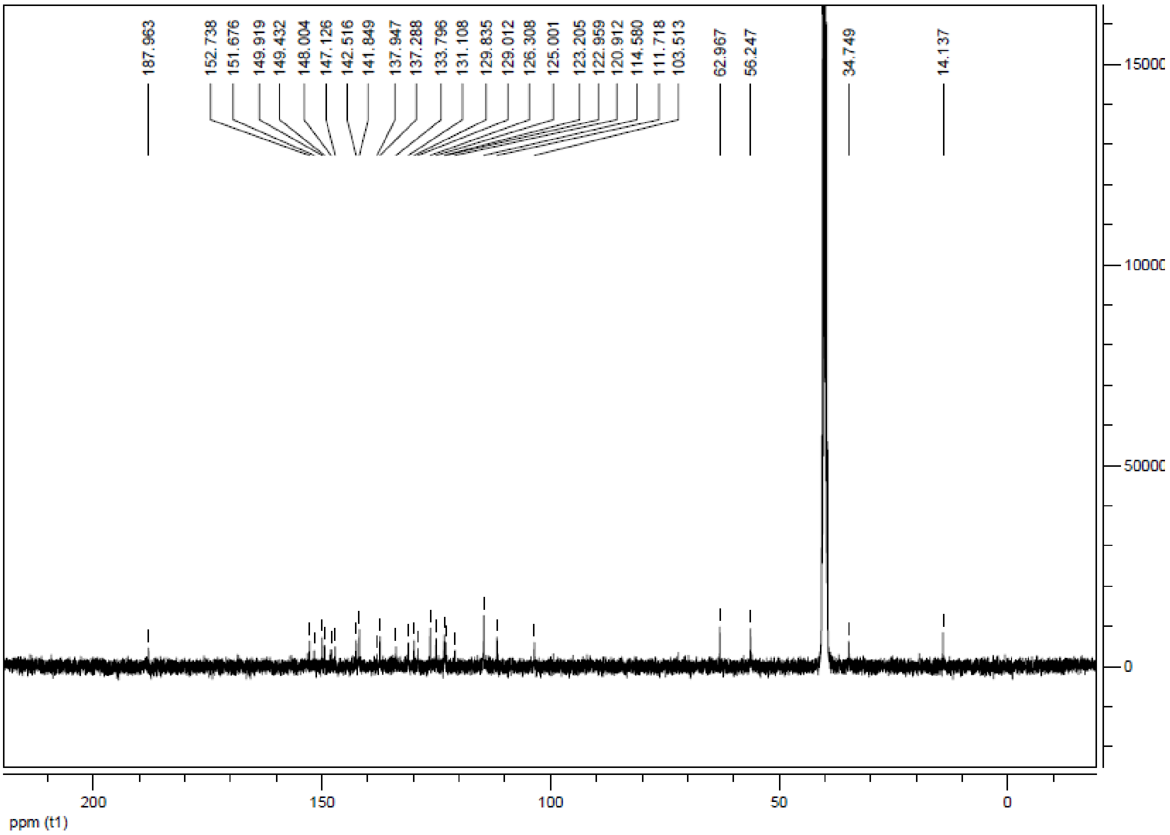

Figure S28. ^{13}C-NMR of compound **7n** (100 MHz, DMSO-*d*6).

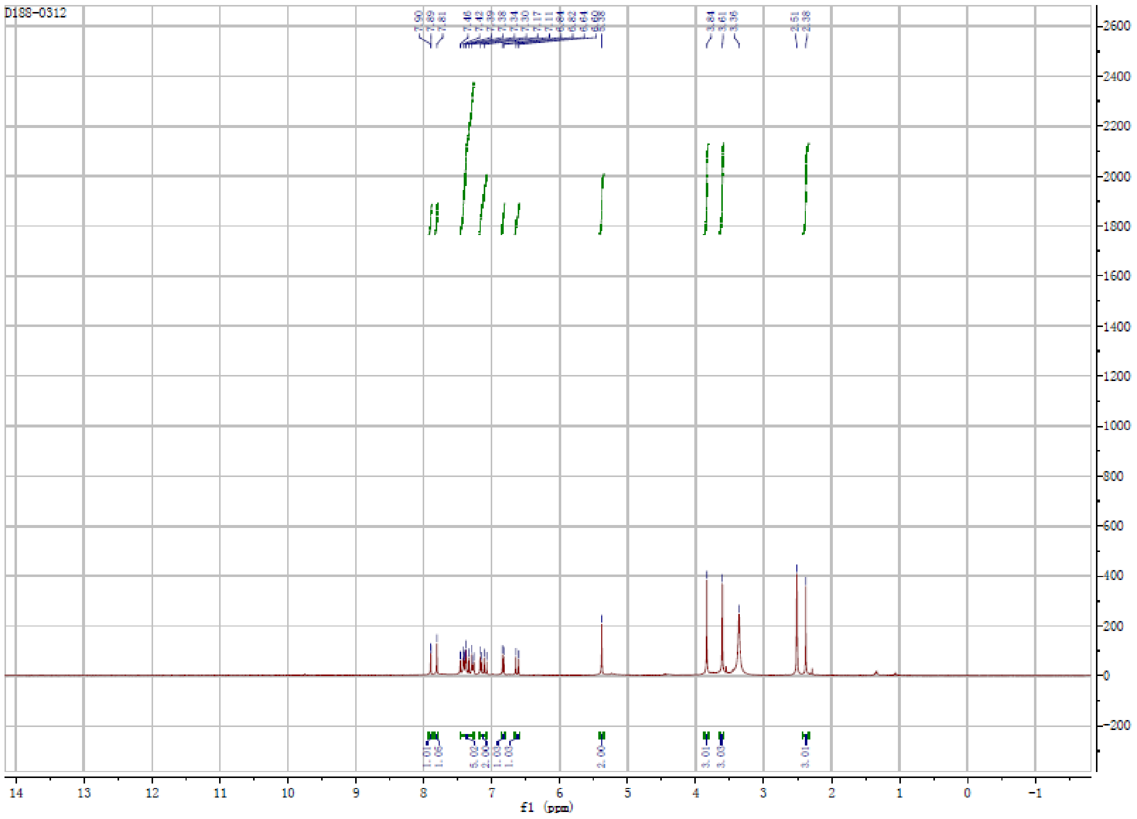

Figure S29. ^{1}H-NMR of compound **7o** (400 MHz, DMSO-d_6).

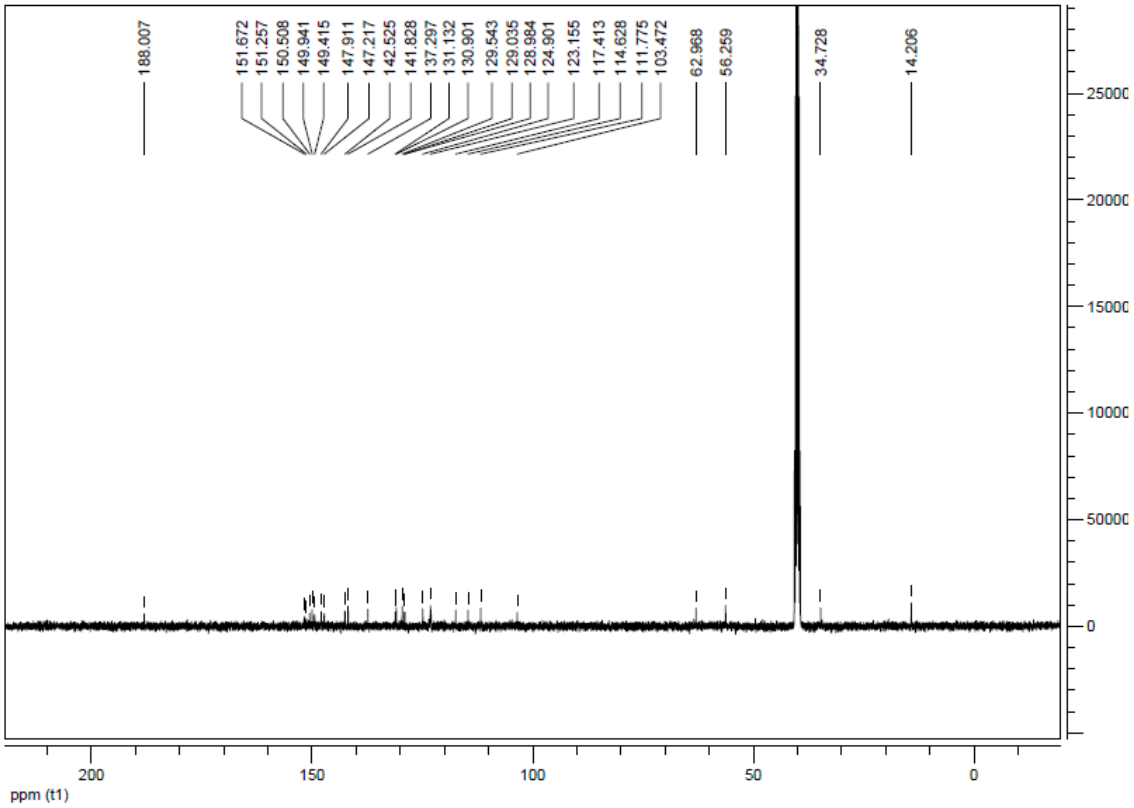

Figure S30. ^{13}C-NMR of compound **7o** (100 MHz, DMSO-d_6).

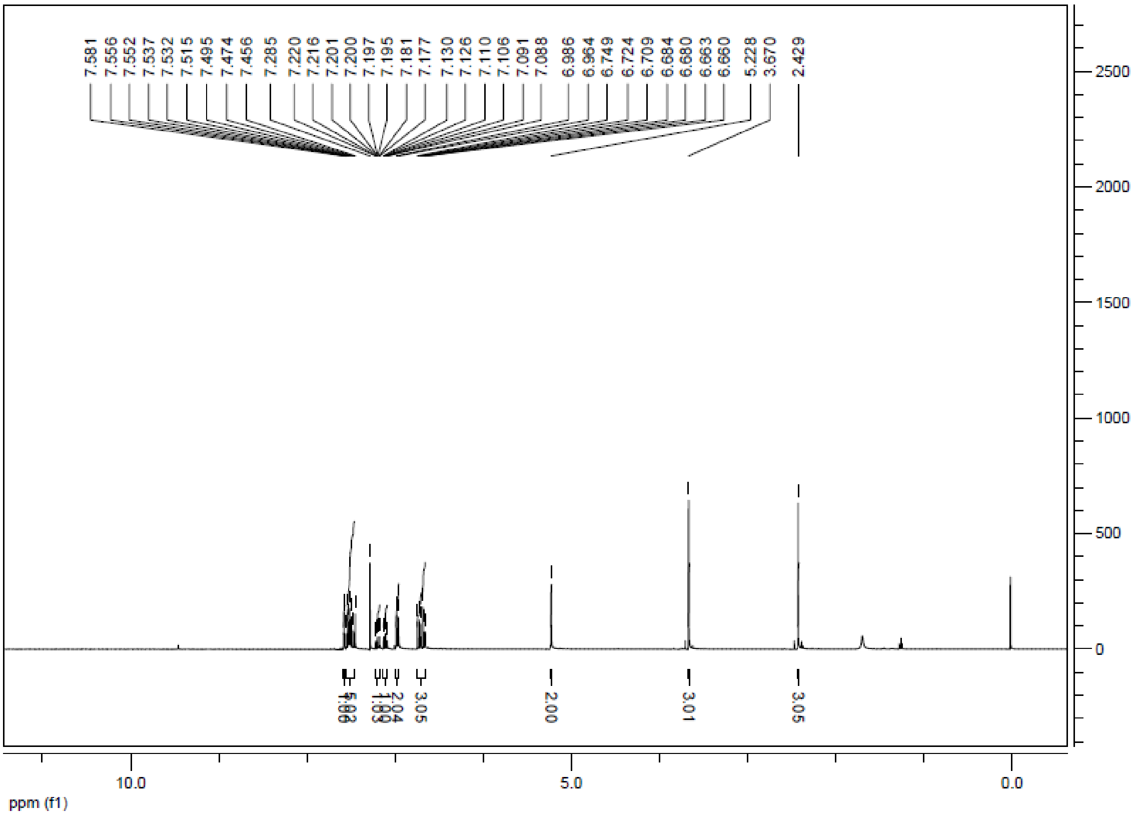

Figure S31. ^{1}H-NMR of compound **7p** (400 MHz, $CDCl_3$).

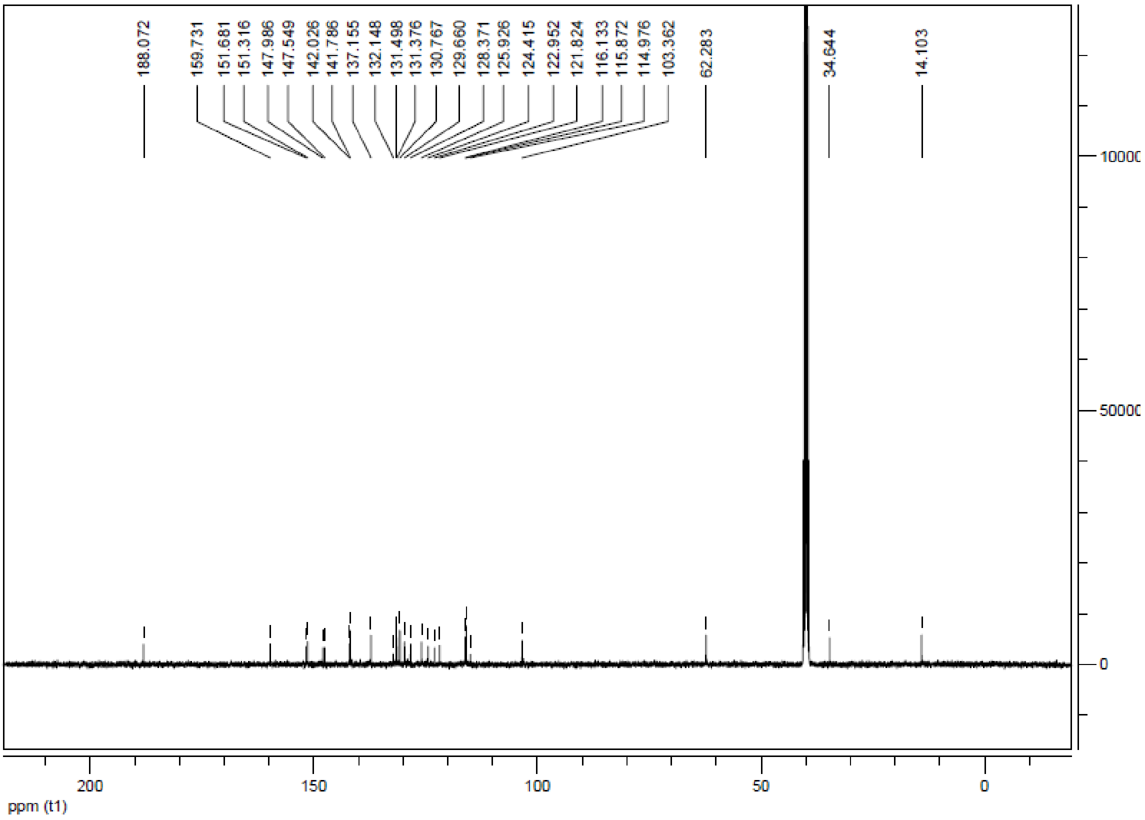

Figure S32. ^{13}C-NMR of compound **7p** (100 MHz, DMSO-d_6).

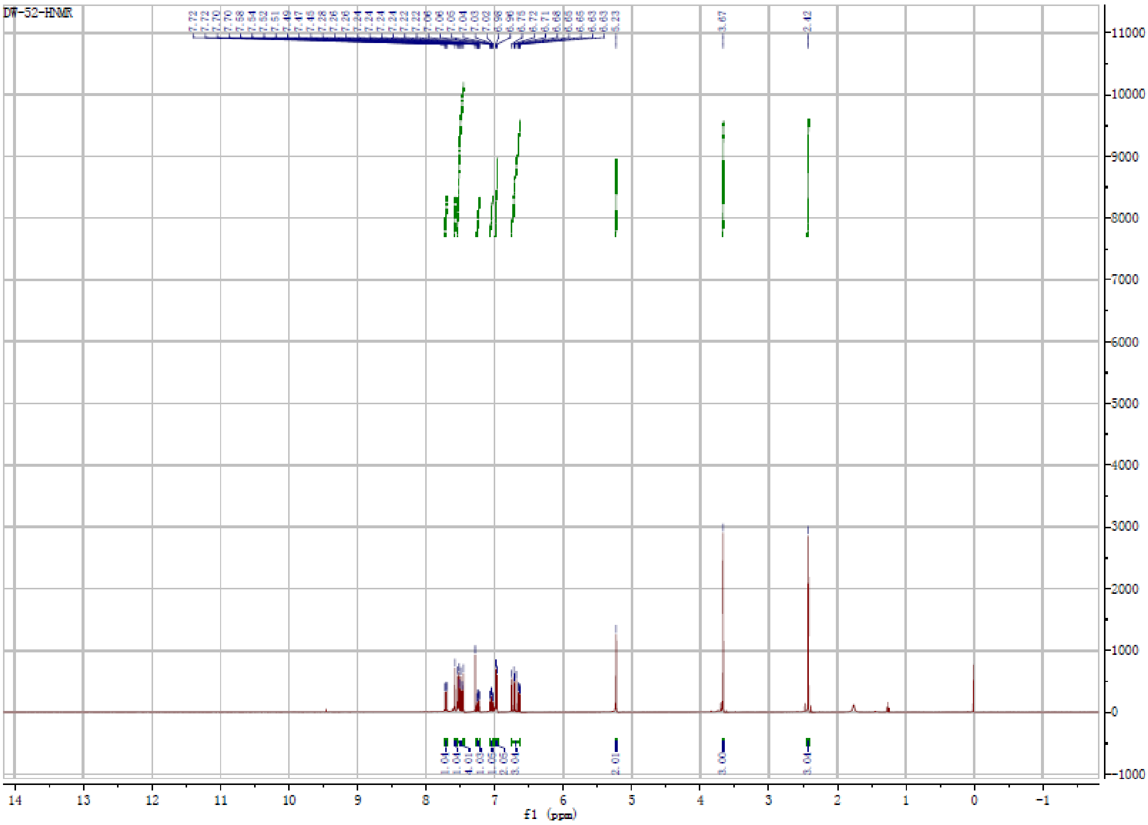

Figure S33. ^{1}H-NMR of compound **7q** (400 MHz, $CDCl_3$).

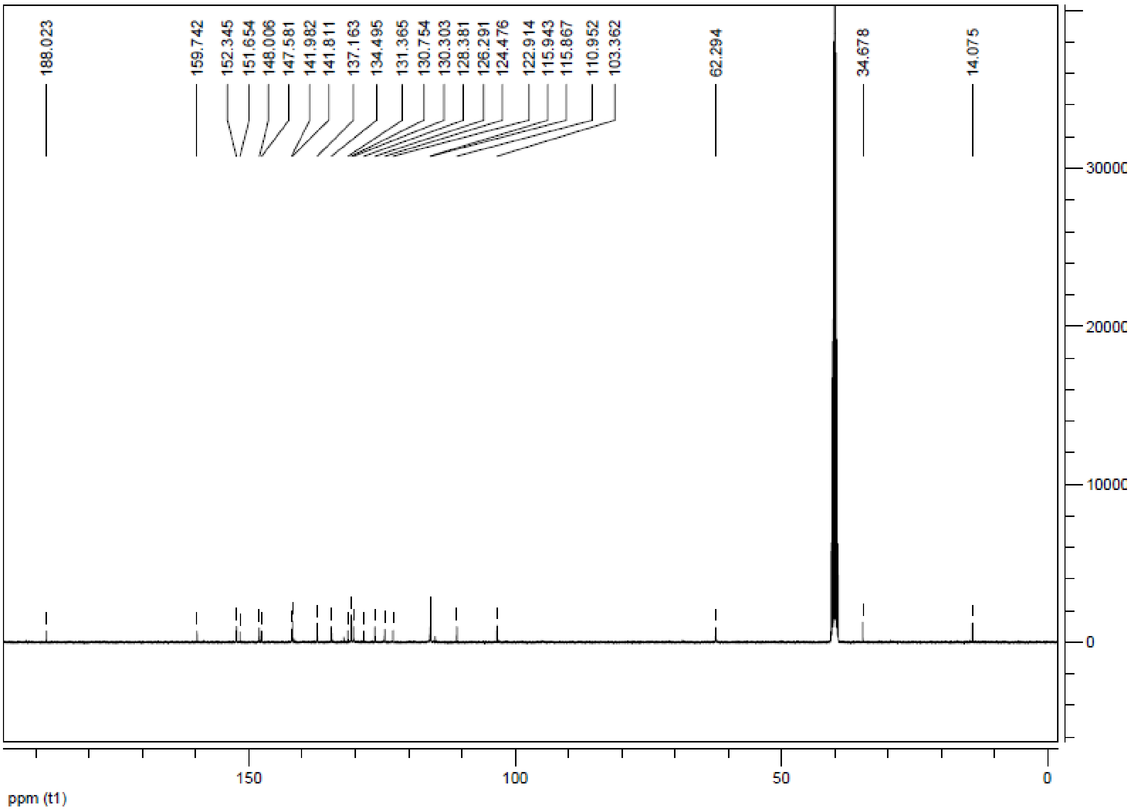

Figure S34. ^{13}C-NMR of compound **7q** (100 MHz, DMSO-d_6).

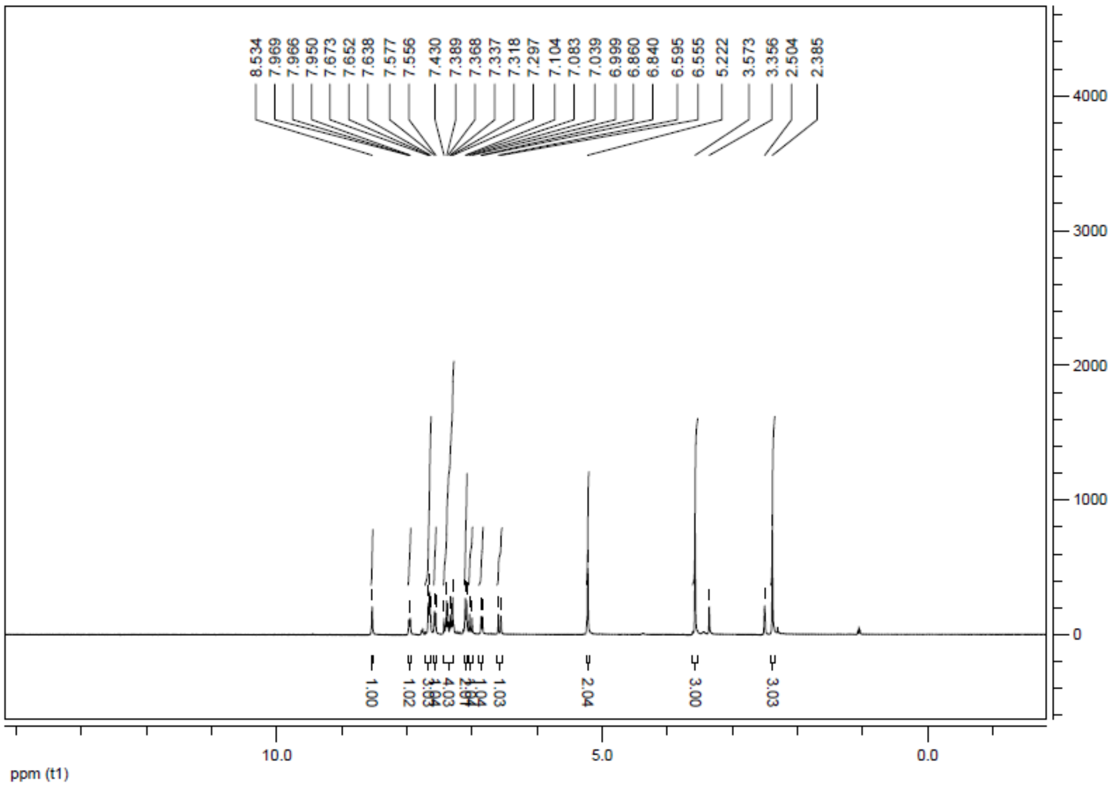

Figure S35. ^{1}H-NMR of compound **7r** (400 MHz, DMSO-d_6).

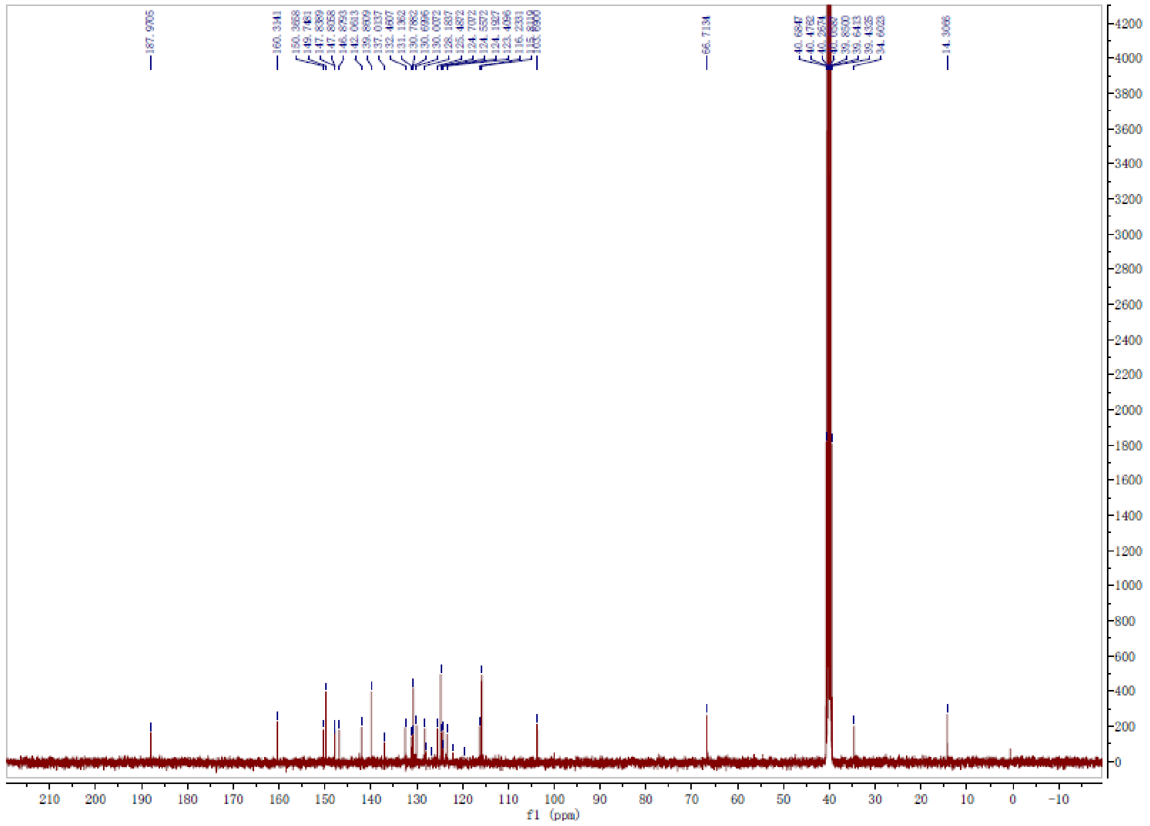

Figure S36. ^{13}C-NMR of compound **7r** (100 MHz, DMSO-d_6).

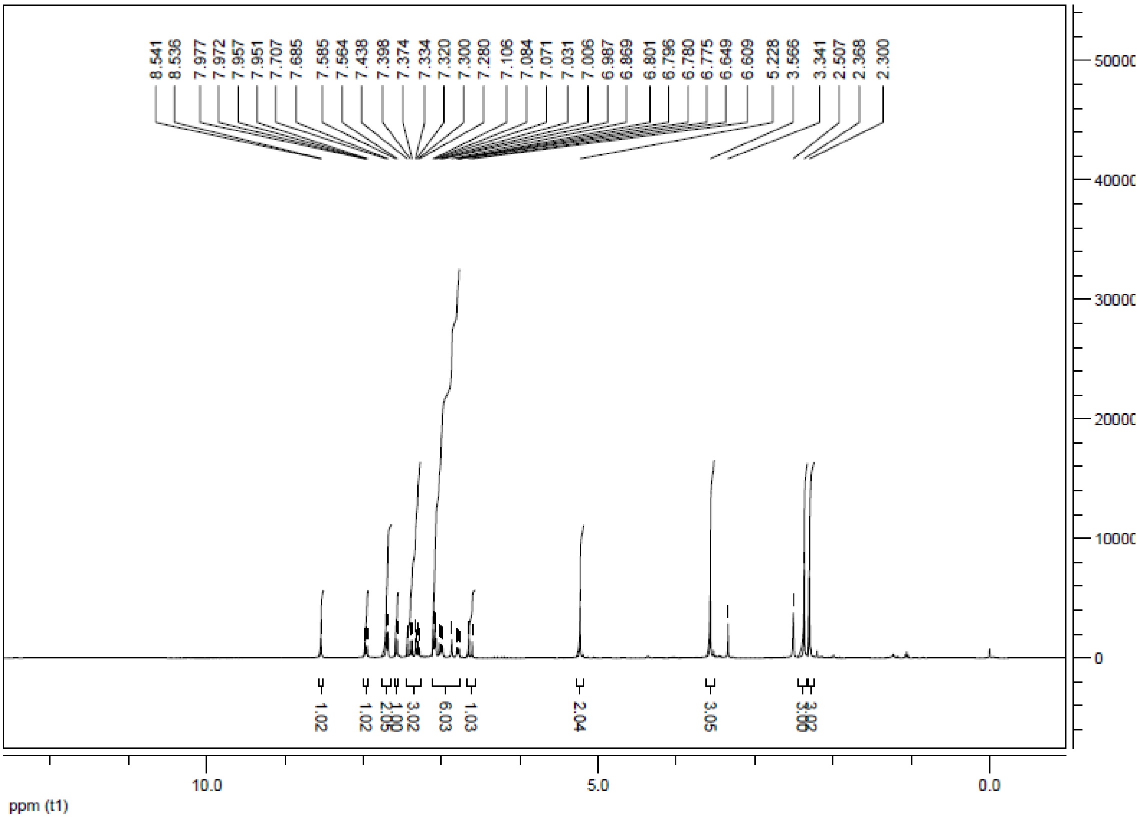

Figure S37. ^{1}H-NMR of compound **7s** (400 MHz, DMSO-*d*$_6$).

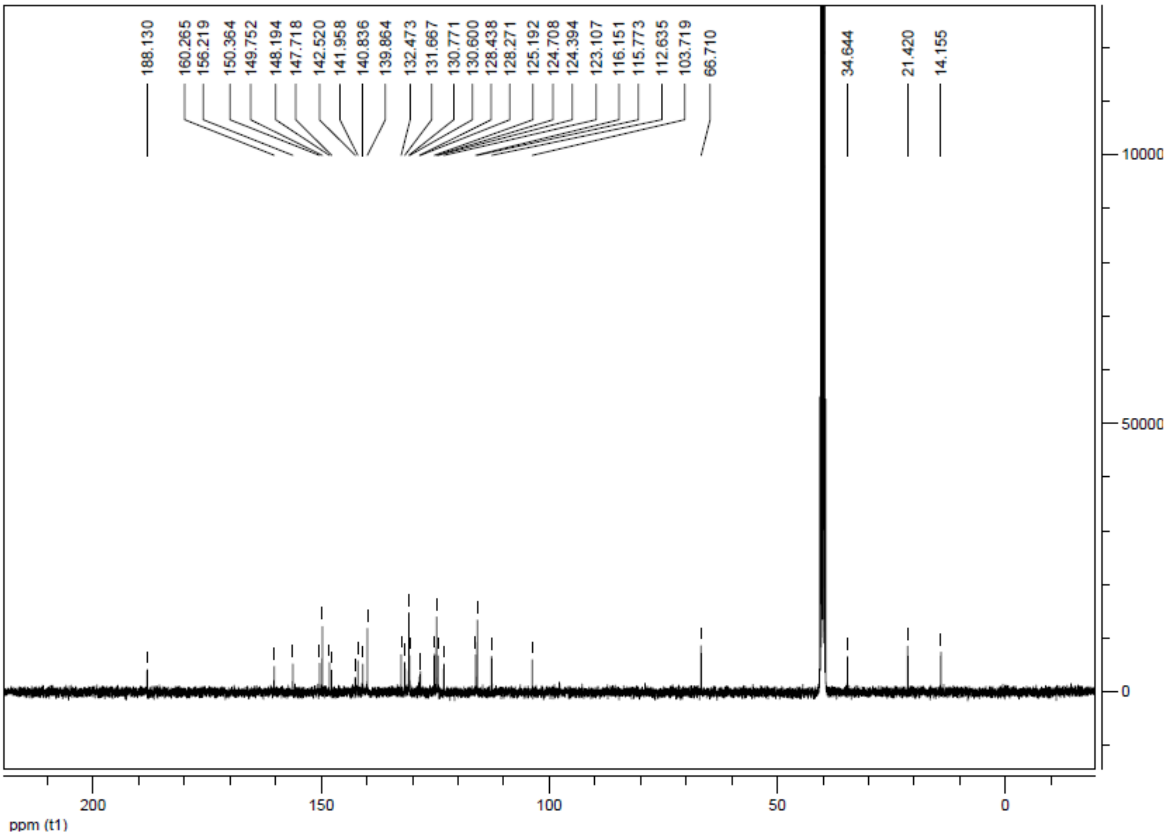

Figure S38. ^{13}C-NMR of compound **7s** (100 MHz, DMSO-*d*$_6$).

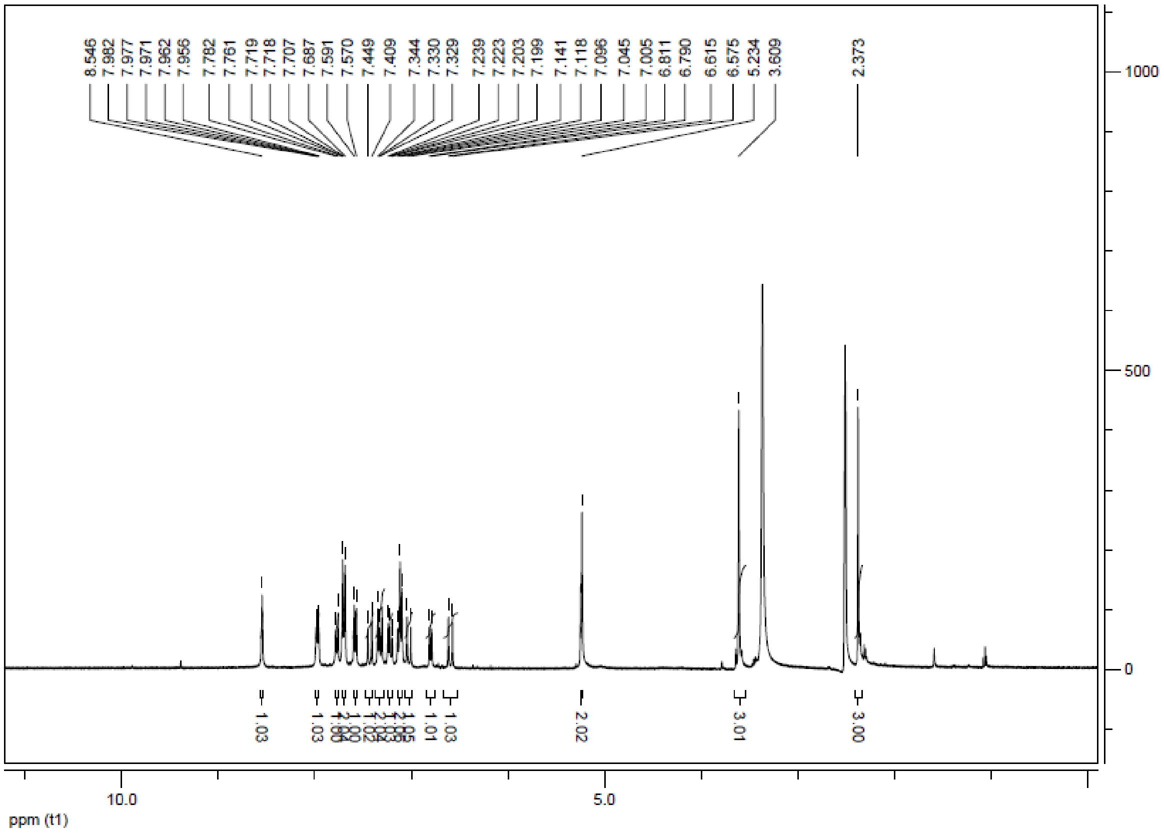

Figure S39. ^{1}H-NMR of compound **7t** (400 MHz, DMSO-*d*6).

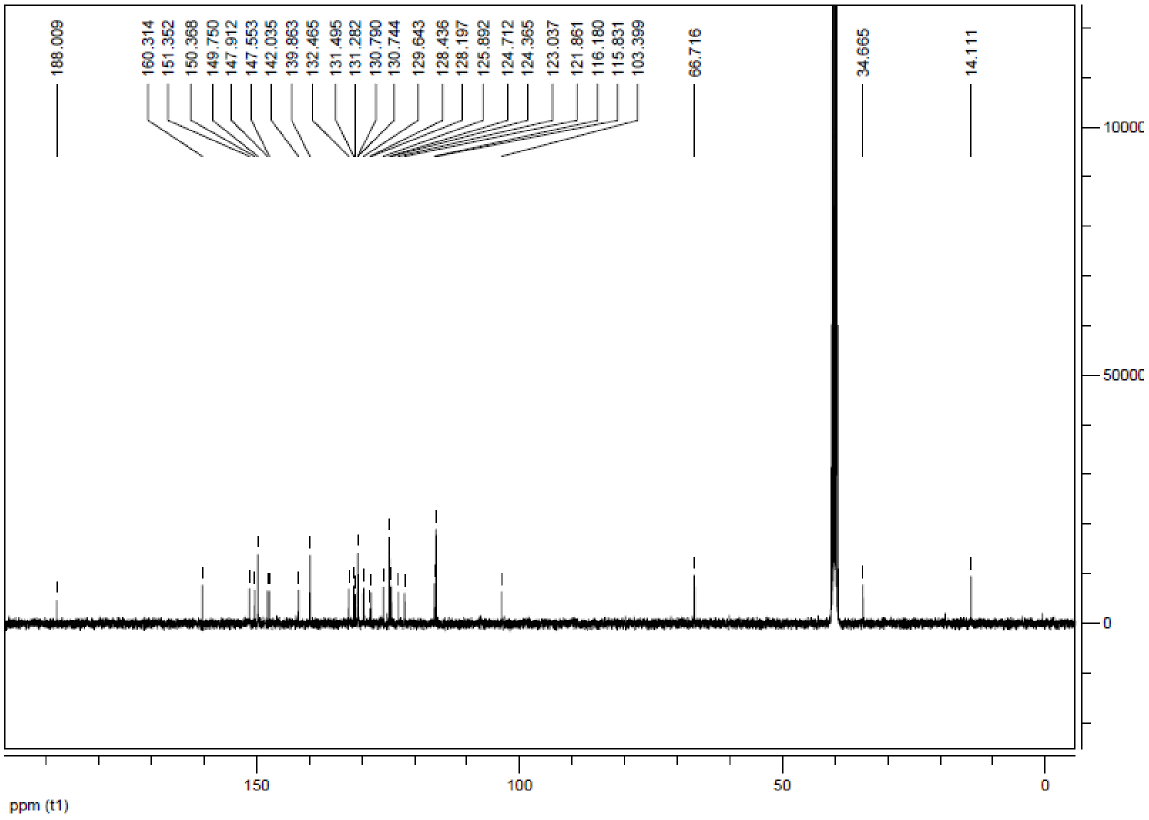

Figure S40. ^{13}C-NMR of compound **7t** (100 MHz, DMSO-*d*6).

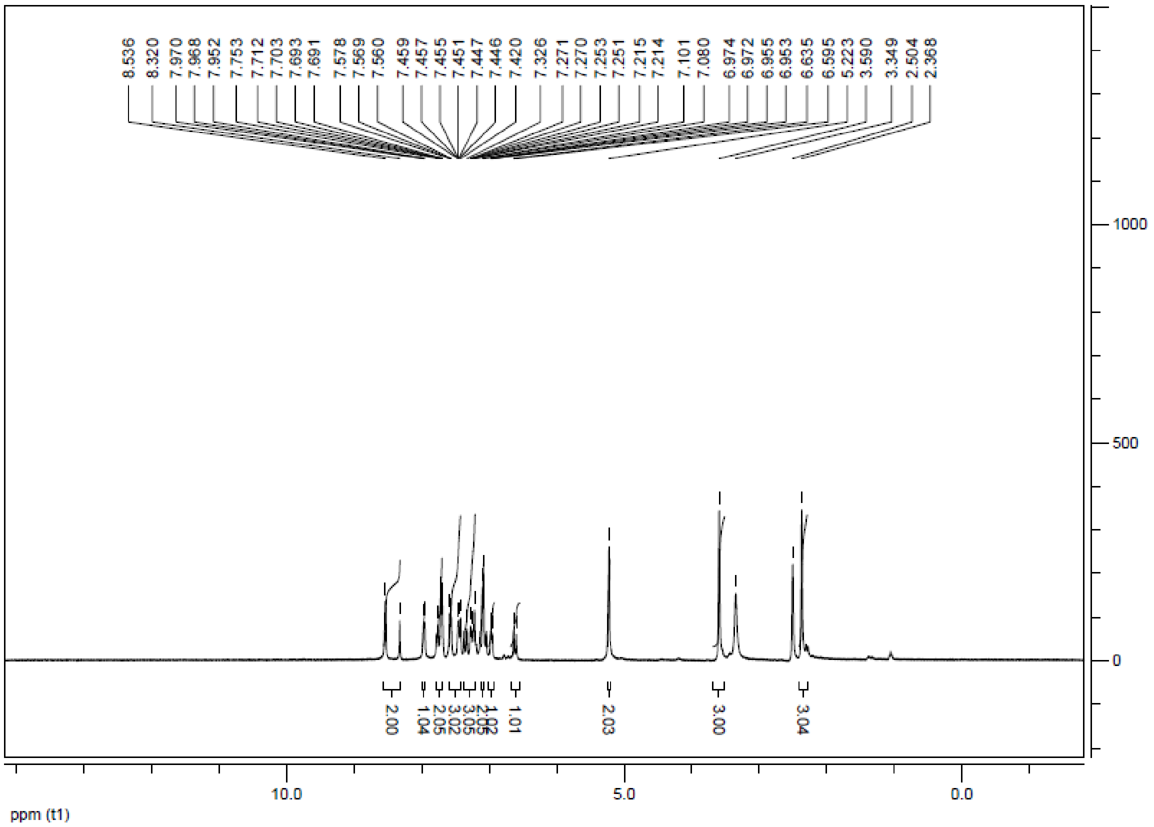

Figure S41. ^{1}H-NMR of compound **7u** (400 MHz, DMSO-d_6).

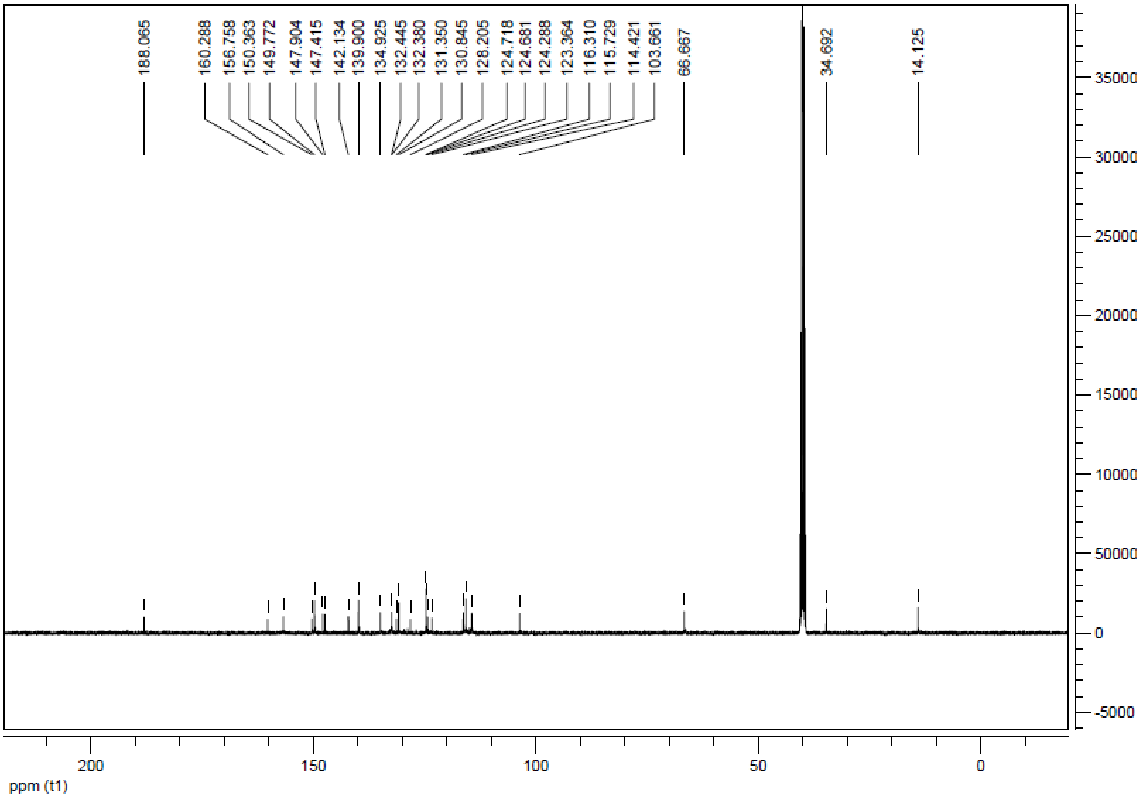

Figure S42. ^{13}C-NMR of compound **7u** (100 MHz, DMSO-d_6).

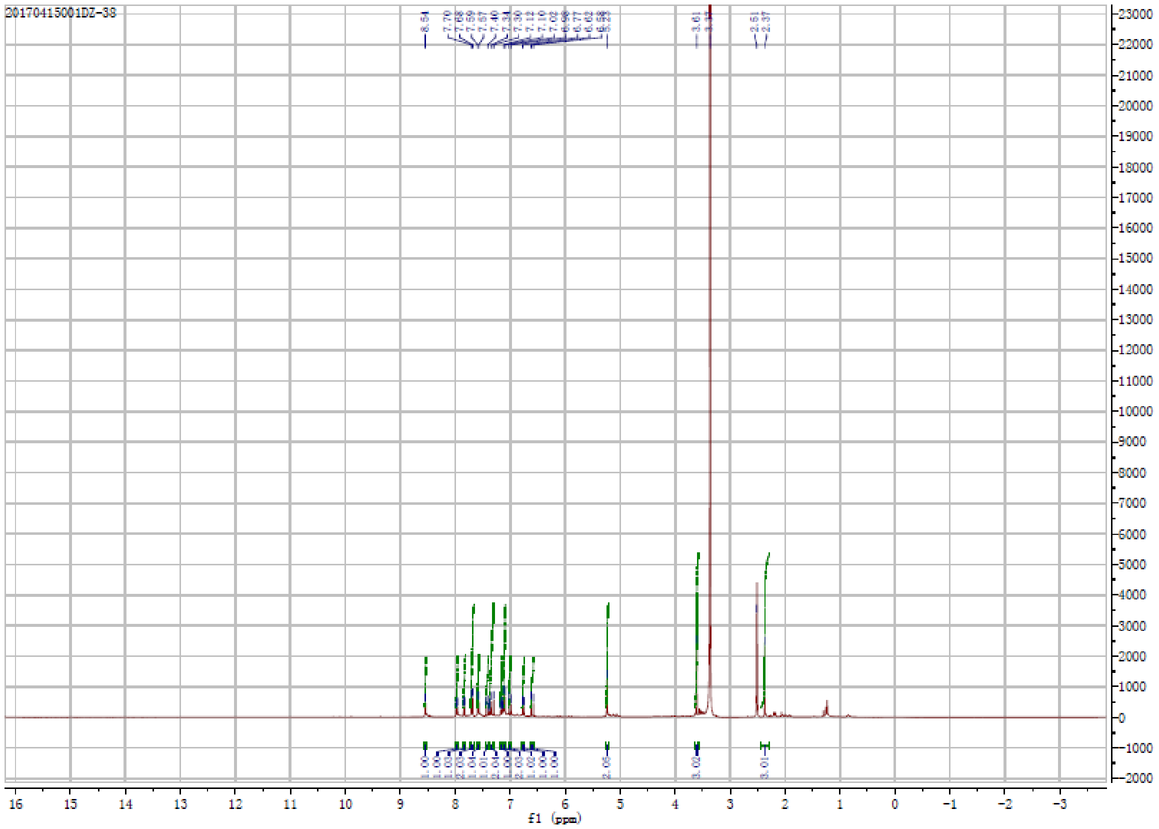

Figure S43. ^{1}H-NMR of compound **7v** (400 MHz, DMSO-d_6).

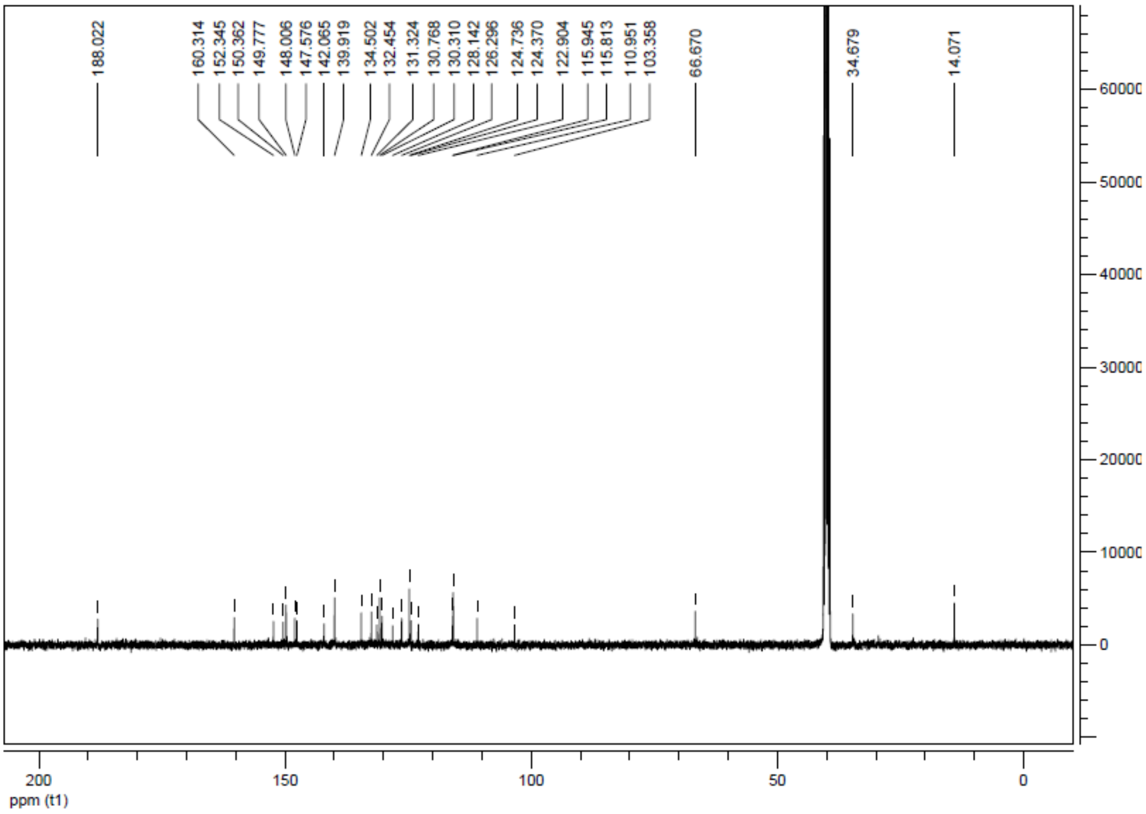

Figure S44. ^{13}C-NMR of compound **7v** (100 MHz, DMSO-d_6).

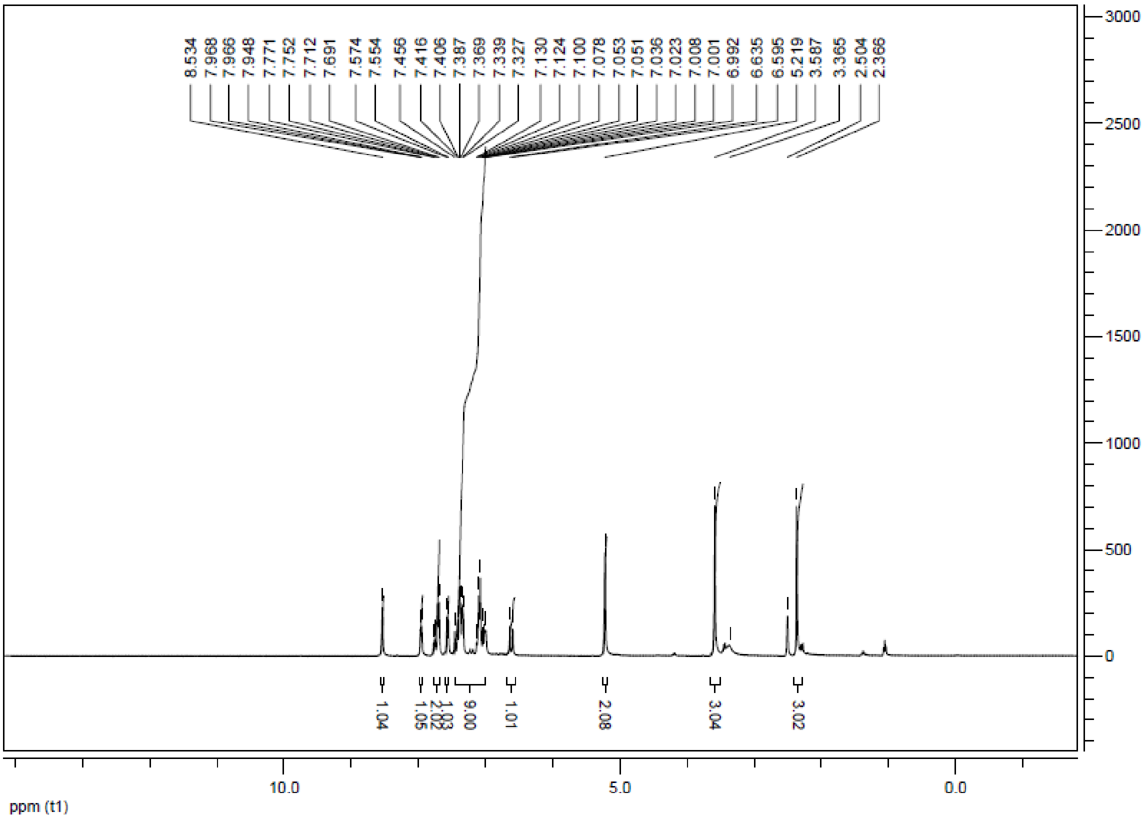

Figure S45. ^{1}H-NMR of compound **7w** (400 MHz, DMSO-*d*6).

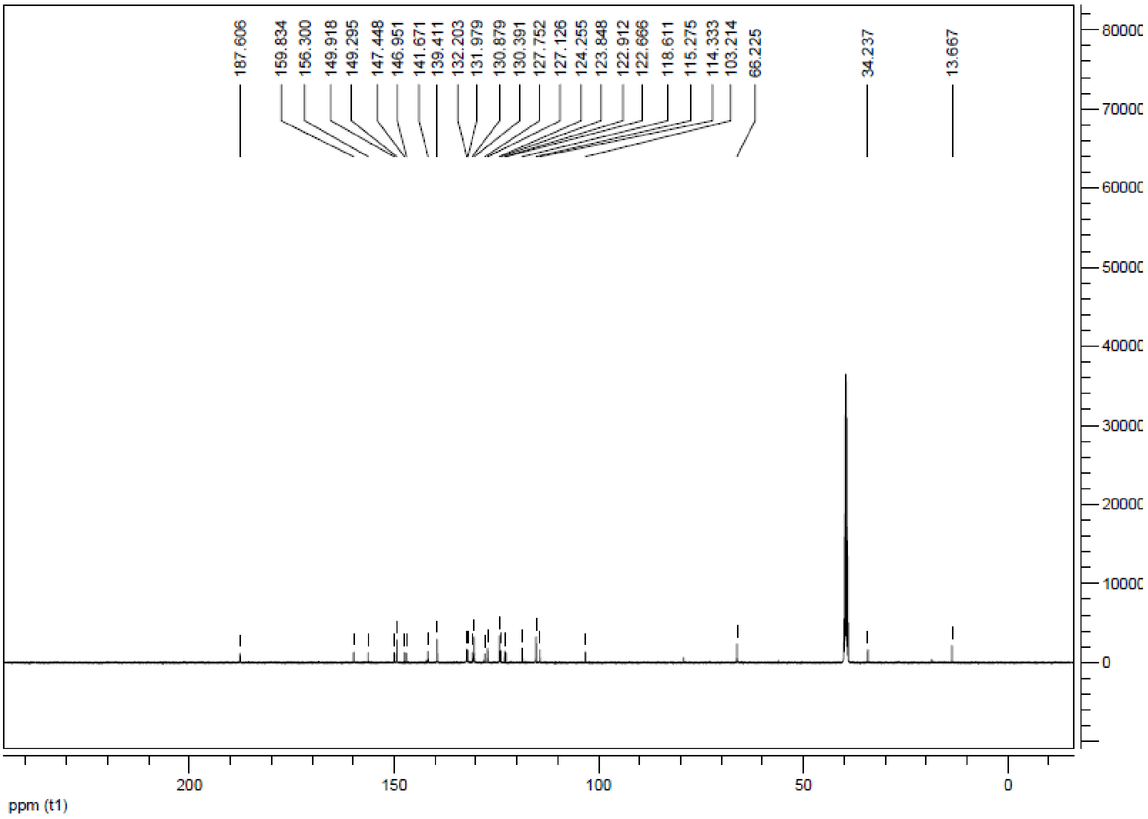

Figure S46. ^{13}C-NMR of compound **7w** (100 MHz, DMSO-*d*6).

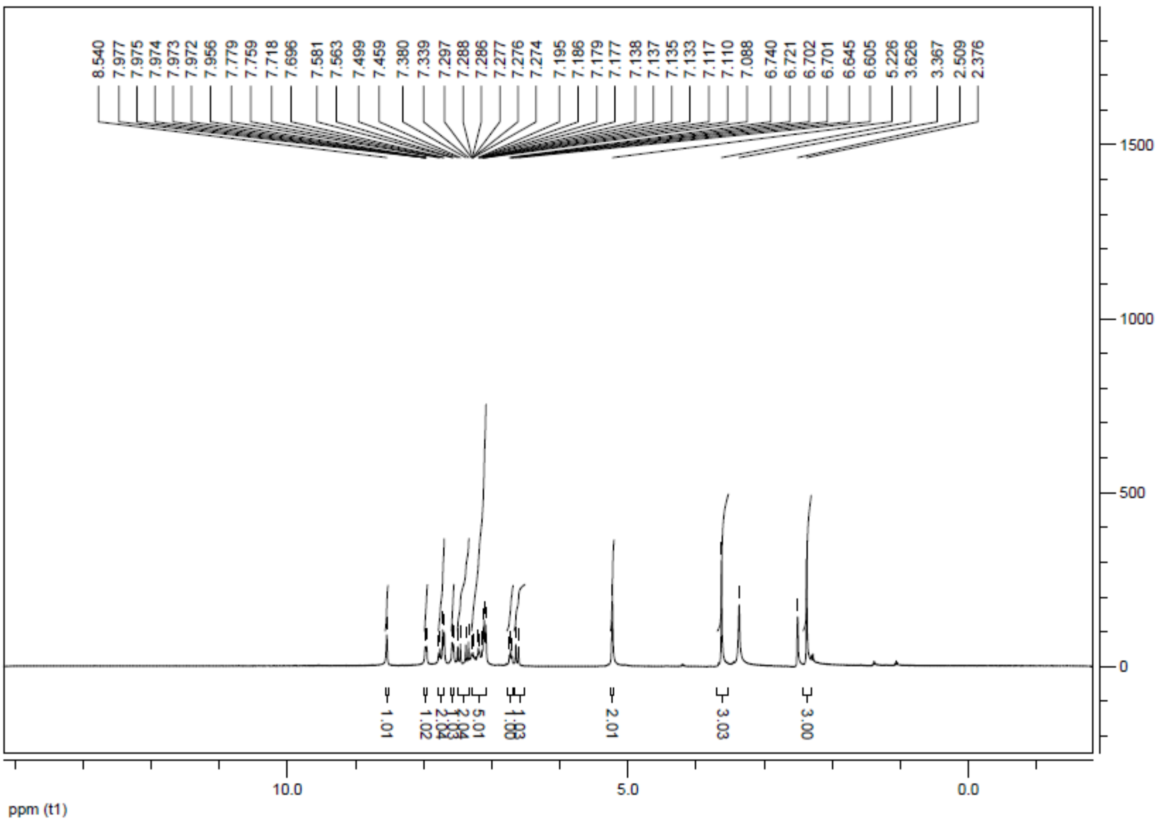

Figure S47. ^{1}H-NMR of compound **7x** (400 MHz, DMSO-*d*6).

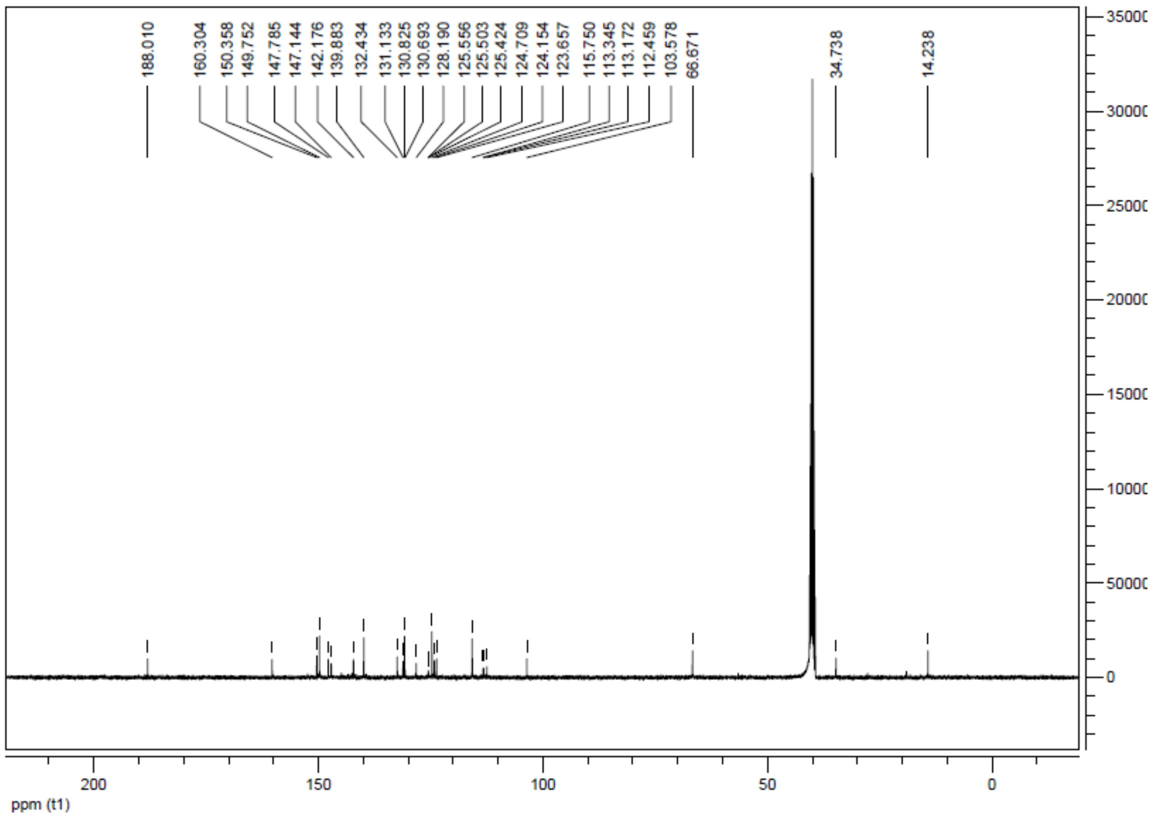

Figure S48. ^{13}C-NMR of compound **7x** (100 MHz, DMSO-*d*6).

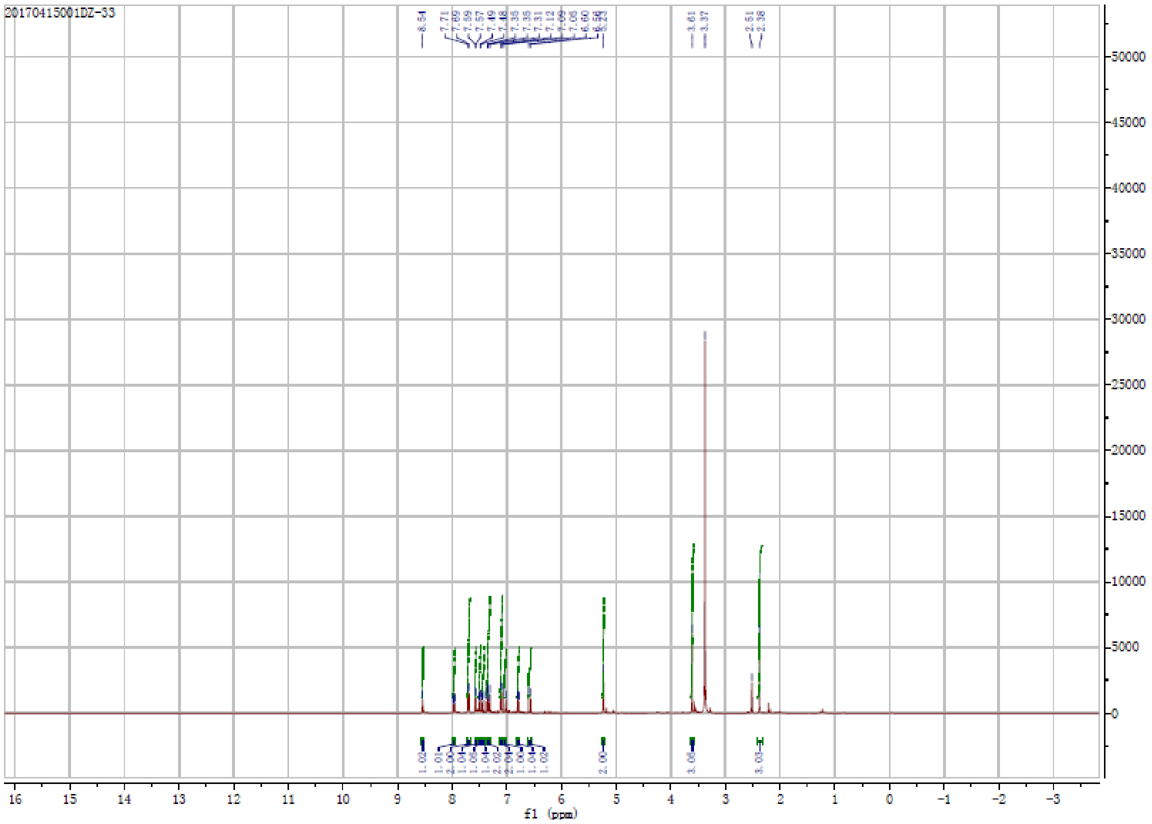

Figure S49. ^{1}H-NMR of compound **7y** (400 MHz, DMSO-d_6).

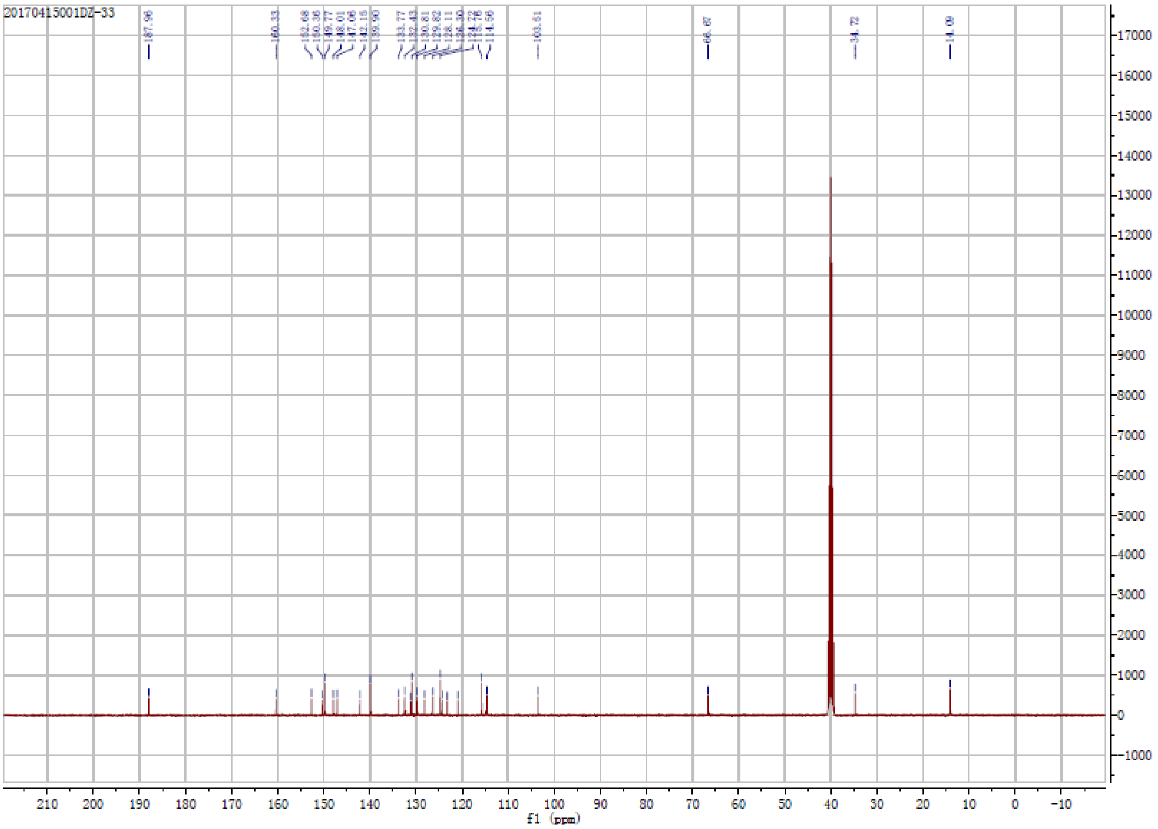

Figure S50. ^{13}C-NMR of compound **7y** (100 MHz, DMSO-d_6).

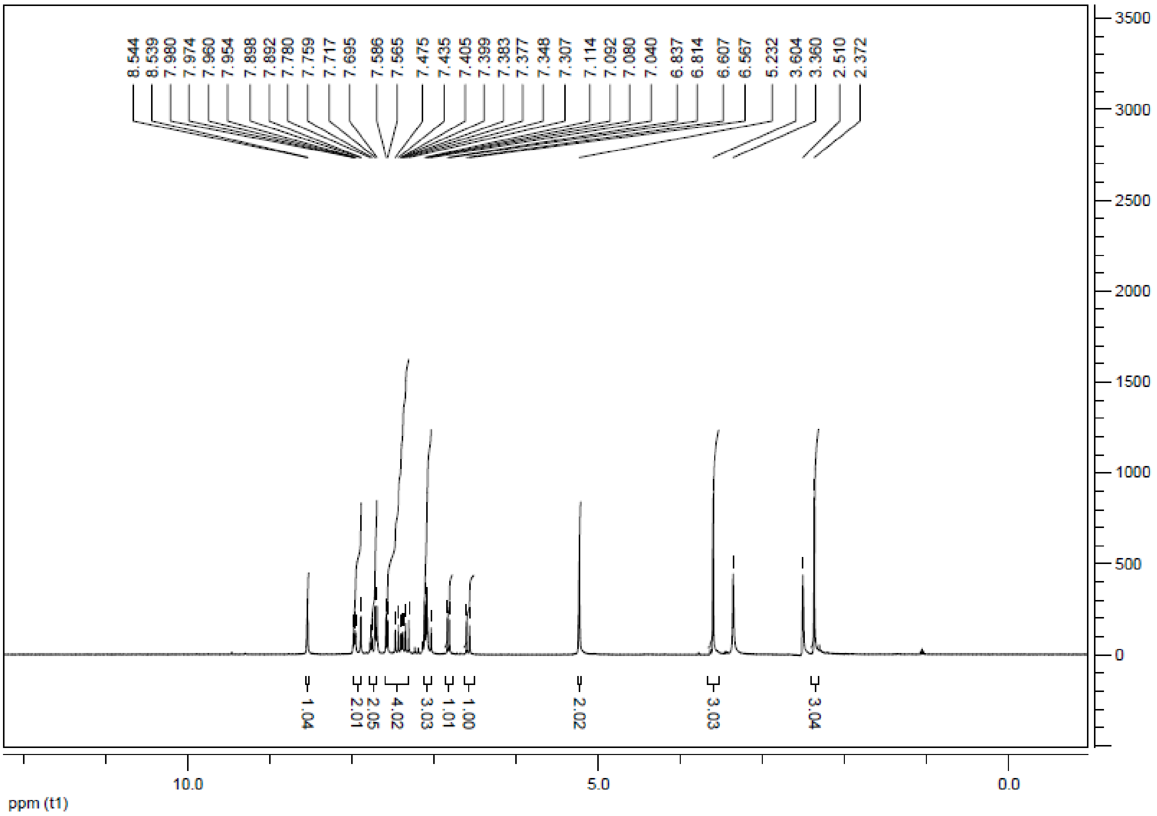

Figure S51. ^{1}H-NMR of compound **7z** (400 MHz, DMSO-*d*$_6$).

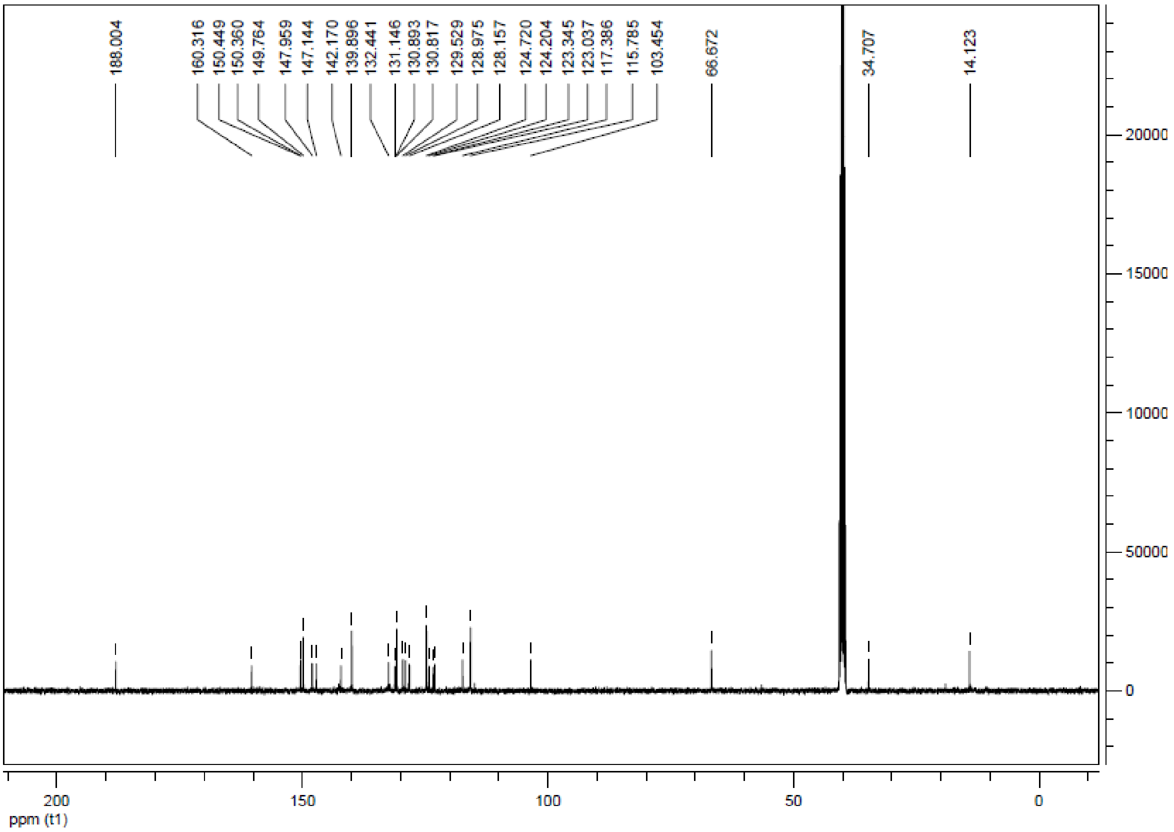

Figure S52. ^{1}H-NMR of compound **7z** (400 MHz, DMSO-*d*$_6$).
